# Self-Assembled Nanomaterials Based on Complementary Sn(IV) and Zn(II)-Porphyrins, and Their Photocatalytic Degradation for Rhodamine B Dye

**DOI:** 10.3390/molecules26123598

**Published:** 2021-06-11

**Authors:** Nirmal K. Shee, Hee-Joon Kim

**Affiliations:** Department of Applied Chemistry, Kumoh National Institute of Technology, Gumi 39177, Korea; nirmalshee@gmail.com

**Keywords:** complementary porphyrins, self-assembly, nanomaterial, photocatalytic degradation

## Abstract

A series of porphyrin triads (**1**–**6**), based on the reaction of *trans*-dihydroxo-[5,15-bis(3-pyridyl)-10,20-bis(phenyl)porphyrinato]tin(IV) (**SnP**) with six different phenoxy Zn(II)-porphyrins (**ZnL^n^**), was synthesized. The cooperative metal–ligand coordination of 3-pyridyl nitrogens in the **SnP** with the phenoxy Zn(II)-porphyrins, followed by the self-assembly process, leads to the formation of nanostructures. The red-shifts and remarkable broadening of the absorption bands in the UV–vis spectra for the triads in CHCl_3_ indicate that nanoaggregates may be produced in the self-assembly process of these triads. The emission intensities of the triads were also significantly reduced due to the aggregation. Microscopic analyses of the nanostructures of the triads reveal differences due to the different substituents on the axial Zn(II)-porphyrin moieties. All these nanomaterials exhibited efficient photocatalytic performances in the degradation of rhodamine B (RhB) dye under visible light irradiation, and the degradation efficiencies of RhB in aqueous solution were observed to be 72~95% within 4 h. In addition, the efficiency of the catalyst was not impaired, showing excellent recyclability even after being applied for the degradation of RhB in up to five cycles.

## 1. Introduction

Over the past two decades, nanomaterials have drawn the attention of material chemistry researchers due to their extensive applicability in various fields, including catalysis, solar energy conversion and storage, molecular recognition, sensing, and cancer treatment [1,2,3,4,5]. These compounds have been widely used to improve the properties of materials, which include high electrical conductivity, excellent optoelectronics, and corrosion resistance [6,7,8]. There is no doubt that these highly ordered nanostructures exhibit tremendously useful features, including large surface areas, various chemical and physical properties, as well as high thermal stability, compared to their parent components. Therefore, the design and fabrication of these novel nano- and microstructured materials are important to their technological utility [9,10]. A large number of building blocks have been used to endow the desired nanostructures with well-defined shapes and dimensions [11,12,13]. Porphyrin compounds (free porphyrins or metalloporphyrins) are one of the building blocks used for the construction of self-assembled functional nanomaterials that possess a charge-transfer function and enhanced light-harvesting properties [14,15,16,17]. The porphyrinoid compounds form large-scale aggregates in solution via the self-assembly process. The morphology of these structures is not always regular or suitable for many practical applications. Thus, the construction of well-defined nanostructures from the porphyrins with definite sizes, dimensions, and shapes is a formidable assignment. Various intermolecular non-covalent interactions (e.g., hydrogen bonding, ligand coordination, π–π interaction, electrostatics, van der Waals force, as well as hydrophobic and hydrophilic effects) are mainly responsible for the self-assembly of porphyrins [18,19,20,21,22]. Various methods, including re-precipitation, ionic self-assembly, metal–ligand coordination, sonication, and surfactant-assisted methods, have been used for the fabrication of well-defined discrete self-assembled porphyrin nanostructures [23,24,25,26,27].

Nowadays, among other metalloporphyrins, Sn(IV)-porphyrins are widely used as a building block in the construction of functional porphyrin nanoaggregates, including nanotubes, nanofibers, nanosheets, nanocomposites, and nanorods [28,29,30,31,32]. These nanostructures have been widely used for the photocatalytic degradation of organic dyes in an aqueous solution, and the production of hydrogen gas under visible-light irradiation [33,34]. Sn(IV)-porphyrins are ideal scaffolds, as they can readily form stable six-coordinate complexes with two trans-axial oxyanion ligands, such as carboxylates and alkoxides, due to the oxophilic nature of the Sn(IV) center. In addition, these complexes have attractive optical properties and are diamagnetic, meaning that structural information can be obtained from the NMR-active Sn nuclei [35,36,37,38,39].

**Scheme 1:**
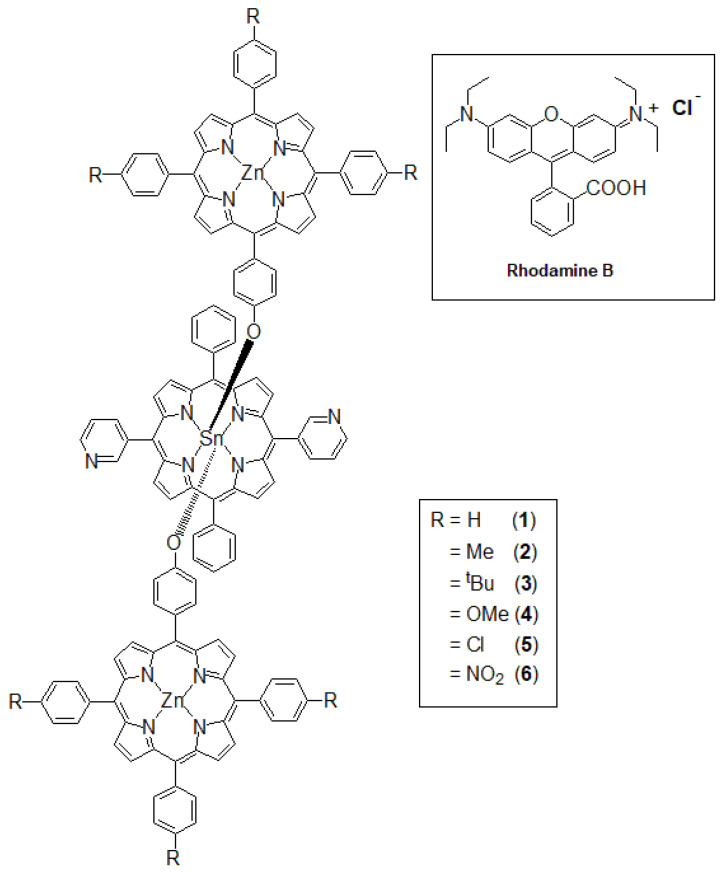
Chemical structures of triads **1**–**6** and dye used in this study.

We are interested in the self-assembly of nanostructures based on combinations of complementary hetero-metalloporphyrins, such as oxophilic Sn(IV)-porphyrins and nitrogen donor-ligating Zn(II)-porphyrins. Recently, we reported that metal–ligand cooperative coordination between a pyridyl Sn(IV)-porphyrin and the phenoxy Zn(II)-porphyrins successfully formed relevant triads, which were easily self-assembled into nanomaterials when in non-coordinating solvents [40]. Here, we present six triads (Scheme 1) obtained from the reaction of *trans*-dihydroxo-[5,15-bis(3-pyridyl)-10,20-bis(phenyl)porphyrinato]tin(IV) (**SnP**) with six different phenoxy Zn(II)-porphyrins (**ZnL^n^**). We explored the self-assembled nanostructures of these triads with various substituents and utilized these materials for the photocatalytic degradation of organic dye rhodamine B in an aqueous solution.

## 2. Results and Discussion

### 2.1. Syntheses

**Scheme 2:**
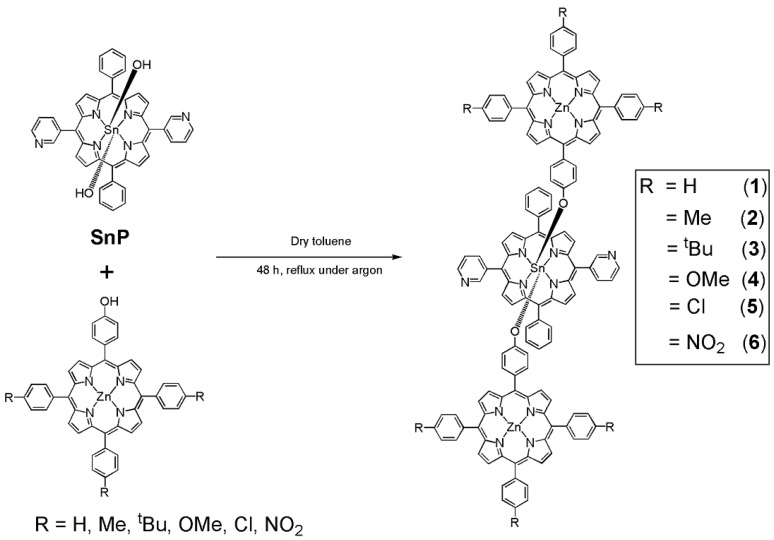
Scheme for the synthesis of triads **1**–**6**.

We used the complementary coordination approach for the synthesis of these triads (Scheme 2). The strong tendency of Sn(IV)-porphyrin to form bonds with aryloxides drove the formation of stable porphyrin arrays [40,41,42,43]. All six triads were synthesized by refluxing corresponding Zn(II) complexes of mono-hydroxyphenyl porphyrin with *trans*-(dihydroxo)Sn(IV)-porphyrins **SnP** in anhydrous toluene for 48 h under an inert Ar atmosphere. The average yields after proper work-up processes were 80% or higher. All the synthesized compounds were fully characterized by various spectroscopic techniques, including elemental analysis, ^1^H-NMR, UV–vis spectroscopy, fluorescence spectroscopy, ESI-mass spectrometry, and scanning electron microscopy (SEM).

### 2.2. Spectroscopic Characterization

The ^1^H-NMR spectra of all six diamagnetic triads (**1**–**6**) along with the individual unconnected monomers (free and Zn(II) porphyrin complexes) are shown in the Supplementary Materials (Figures S1–S18). From the ^1^H-NMR spectra of six triads, it is clear that the resonance positions of the β-pyrrolic protons or the aromatic protons of the central Sn(IV)-porphyrin are very similar to those of the starting **SnP** [Sn(OH)_2_(DPyDPP)] [40,41]. The β-pyrrolic protons of the triads appeared between 9.05 ppm and 9.17 ppm, whereas the protons at the pyridyl rings (H2,6-Py) appeared at ~9.55 ppm and ~9.28 ppm, respectively. Other aromatic protons of the central Sn(IV)-porphyrin appeared at ~8.10 ppm. On the other hand, due to the strong ring current effect of the central Sn(IV)-porphyrins, the resonance positions and the splitting patterns of the axial Zn(II)-porphyrins were different from those of the monomeric Zn(II)-porphyrins. The aryloxy protons appeared in monomeric Zn(II)-porphyrins at ~7.17 and ~7.96 ppm (except for **ZnL^6^**, which appeared at ~7.23 and 8.20 ppm) as two set of doublets, respectively. When in triads, these two protons experience a strong shielding effect caused by the ring current of the central Sn(IV)-porphyrins. These protons resonated as two doublets at 2.62–2.70 and 6.65–6.80 ppm, respectively. The Δδ values (i.e., δ(**ZnL^n^**)–δ(triad)) of these protons were ~4.60 and ~1.30 ppm, respectively. A similar kind of shielding effect was observed for the β–pyrrole protons of the axial porphyrins unit in triads compared to that seen in the monomeric Zn(II) complexes. The signals for the β–pyrrole protons of the monomeric Zn(II)-porphyrins appeared at ~8.8 ppm and ~8.9 ppm. These protons in the triads were shifted to the upfield zone and split into a singlet at ~ 8.75 ppm, along with a pair of doublets at ~8.55 and ~8.40 ppm, respectively. The ^1^H-NMR technique considers the interaction of the protons in axial porphyrins with the central Sn(IV)-porphyrin, and manifests the ring current-induced shifts and resonance couplings [40,41]. As such, this method has been extensively used in the literature to explore the axial bonding-type architecture of similar compounds [40,41,42,43]. The electrospray ionization mass spectra (ESI-MS) of all the six triads are shown in Supplementary Figures S19–S24. For each triad, we observed parent peaks at 769.13, corresponding to [SnP(H)]^+^. However, a low-intensity molecular ion peak was also found for all six triads. It is thus clear that all the compounds were fragmented during the mass spectrometry experiments [40].

**Figure 1:**
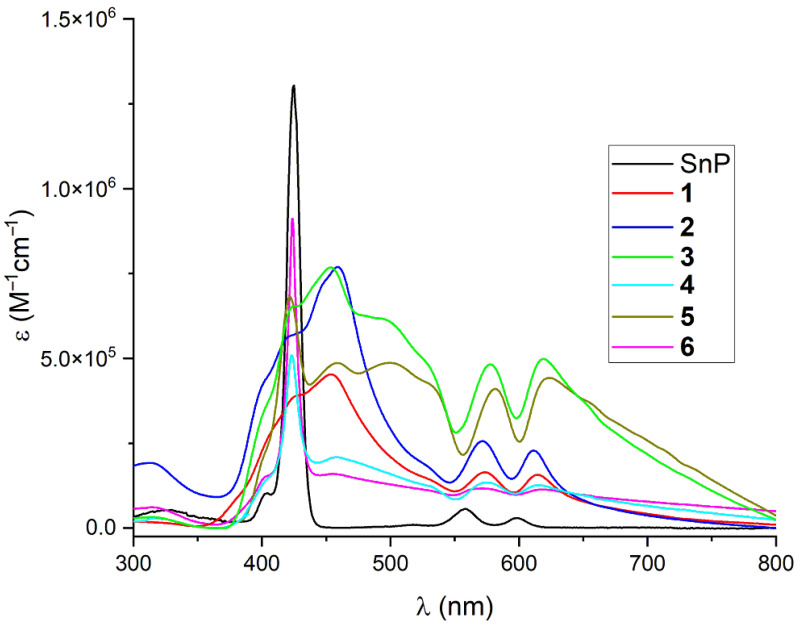
UV–visible spectra of triads **1**–**6** and **SnP** in CHCl_3_.

The UV–visible spectra of all six triads in the CHCl_3_ solution are depicted in Figure 1, along with an **SnP** spectrum for comparison. The peak positions, molar extinction coefficients (*ε*), and maximum absorbance (*λ*_max_) of all compounds, including monomeric Zn(II)-porphyrins, are reported in the experimental section.

All the monomeric Zn(II)-porphyrins showed a Soret band at ~421 nm and Q-bands at ~548 and ~586 nm. The **SnP** also exhibited a Soret band at ~425 nm and Q-bands at ~558 and ~598 nm. The spectra obtained for the triads are different. Generally, the molar extinction coefficient, as well as the peak position (*λ*_max_) values, of these triads are very close to those of the total parent monomeric porphyrin units [40,41,42,43]. In general, these types of compounds show minimal perturbation in response to the minimal interactions between the electronic structures of the discrete macrocyclic *π*-systems. In our case, these spectra are noticeably broad in the Soret band region (~380 nm to ~550 nm), with a red-shift (*λ*_max_ ~455 nm) comparable to the corresponding monomeric units. In addition, the Q-bands were red-shifted, with *λ*_max_ values of ~578 nm and ~618 nm. These red-shifts and the noticeable broadening of the absorption bands indicate that aggregation is possible in the self-assembly process of these triads in solution [40]. Due to the different substituents on the monomeric Zn(II)-porphyrins, small variations were observed in the spectral features of these triads.

**Figure 2:**
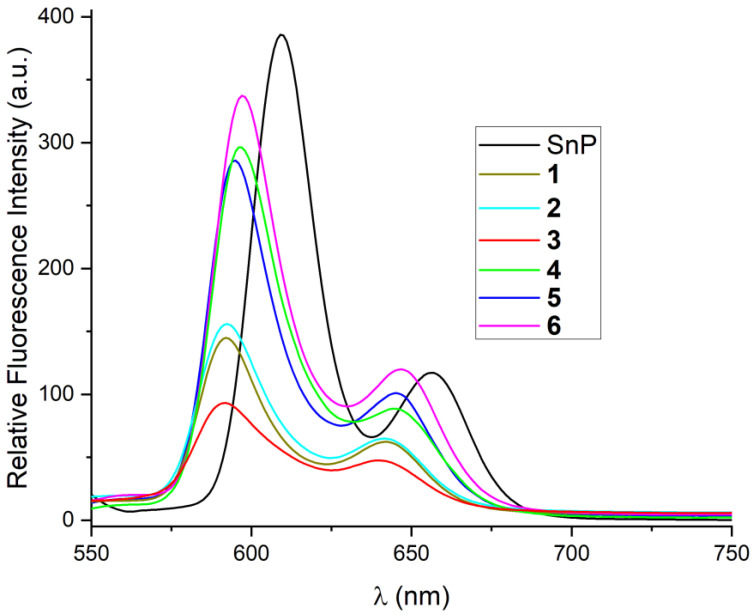
Fluorescence spectra of triads **1**–**6** and **SnP** in CHCl_3_. The optical density (OD) of each sample solution was fixed at 0.20.

The steady-state fluorescence spectra of all six triads and of **SnP** were recorded in CHCl_3_, and are illustrated in Figure 2. The **SnP** gave two-banded fluorescence spectra (*λ*_ext_ = 550 nm), with the emission maxima appearing at 610 and 656 nm. All the triads also exhibited similar two-banded fluorescence spectra (*λ*_ext_ = 550 nm), with the emission maxima appearing at 597 and 647 nm. The peak-to-peak intensity ratio decreased between the **SnP** and the triad. The emission intensity of the triads was significantly reduced due to the aggregation, and the patterns mostly depend on the substituents on the axial porphyrins.

### 2.3. Supramolecular Self-Assembly to Nanostructures

**Figure 3:**
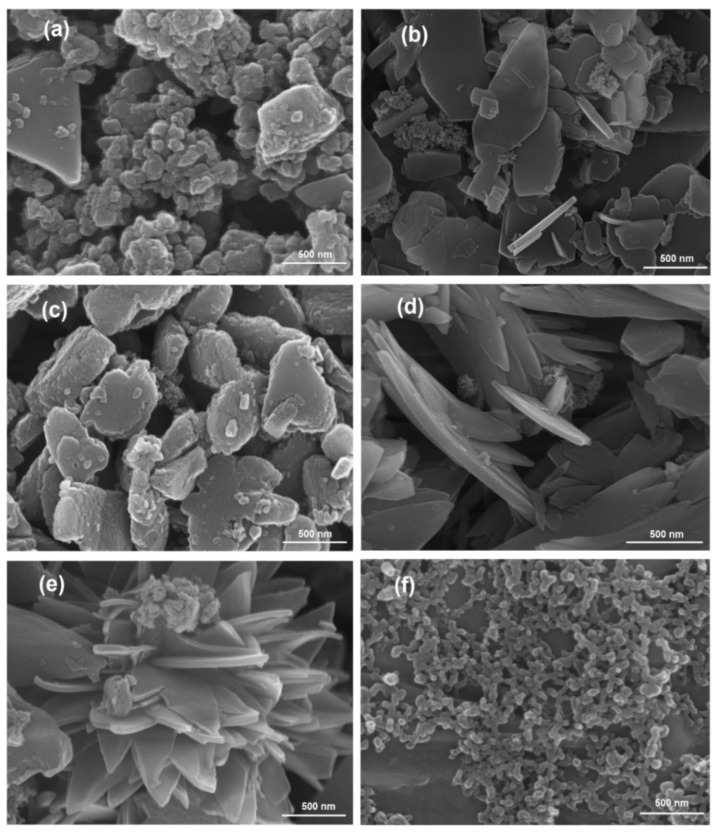
High-resolution FE-SEM (field emission scanning electron microscope) images for the assembly patterns of triads: (**a**) **1**; (**b**) **2**; (**c**) **3**; (**d**) **4**; (**e**) **5**; (**f**) **6**.

The self-assembly identity of all the compounds was analyzed via SEM. For sample preparation, each compound was suspended in toluene at a fixed concentration (*c* = 1 mM) and was centrifuged at 13,500 rpm for 10 s. Then, we drop-cast the solution on the surface of copper tape for deposition, followed by drying in air. After deposition, a Pt coating was required before the SEM could be carried out. All six triads assembled into nanoaggregates and their morphologies are displayed in Figure 3 and Supplementary Figure S25. Nanoflakes of different sizes were observed for triad **1** (Figure 3a and Supplementary Figure S25a). These nearly spherical, spongy nanoflakes were interconnected indiscriminately. The particle sizes were in the range of 100–500 nm (the population of smaller particles was greater than bigger). A mixture of flake-shaped nano-aggregates was also found for triad **2** (Figure 3b and Supplementary Figure S25b), but these particles were separated. The lengths of these flakes ranged from approximately 150 nm to 1 μm, with 50 nm width. In the case of triad **3**, distinct and bigger nanoparticles were formed (Figure 3c and Figure S25c). These stone-like particles were 300~800 nm in length and 150 nm in width. Triad **4** formed a petal-shaped nanostructure with the approximate dimensions of 750 nm to 3 μm, with a 200 nm width (Figure 3d and Supplementary Figure S25d). Flower-shaped nanoaggregates were observed for triad **5** (Figure 3e and Supplementary Figure S25e). Finally, a nearly spherical nanostructure (closely packed) arose in the case of triad **6** (Figure 3f and Supplementary Figure S25f), and these spheres were interconnected. These particles were approximately 80 nm to 150 nm long.

Porphyrin molecules aggregate into nanostructures via self-assembly processes. Various interactions, including hydrogen bonding, π–π stacking, van der Waals forces, and ligand coordination are responsible for this self-assembly process. Previously, we used *meso*-[5-(4-hydroxyphenyl)-10,15,20-tris(3,5-di-*tert*-butylphenyl)porphyrinato]zinc(II) as the source of axial porphyrins, leading to the creation of nanofibers [40]. It has been suggested that the pyridyl groups in Sn(IV)-porphyrin probably coordinated with the Zn ion in the axial Zn(II)-porphyrin intramolecularly. This coordination can prevent the movement of the axial porphyrins, which facilitates the self-assembly processes. All the triad molecules formed nanostructures via π–π stacking interactions between adjacent porphyrin molecules through face-to-face-type interactions. Similarly, we observed different morphologies depending on the substituents on the axial Zn(II)-porphyrins. With varying the substituent at the 4-position from H to Me and again to ^t^Bu, the features of the nanoaggregates were also changed (Figure 3a–c). The nanoparticles were well separated, and their sizes increased. The particle boundaries are clearly visible. It should be also noted that the starting porphyrin monomer, either **SnP** or **ZnL^n^**, displayed no aggregation under the present conditions [40].

On the other hand, triads **4**, **5**, and **6** possessing hetero-atomic functional groups, showed markedly different morphologies from triads **1**, **2**, and **3**. The supramolecular interactions based on their hetero-atomic functional groups may particularly contribute to constructing the nanostructured porphyrin assemblies. The halogen–halogen interaction has been recently studied in the self-assembly of metalloporphyrins [44].

### 2.4. Photocatalysis for the Degradation of Rhodamine B (RhB) Dye

**Figure 4:**
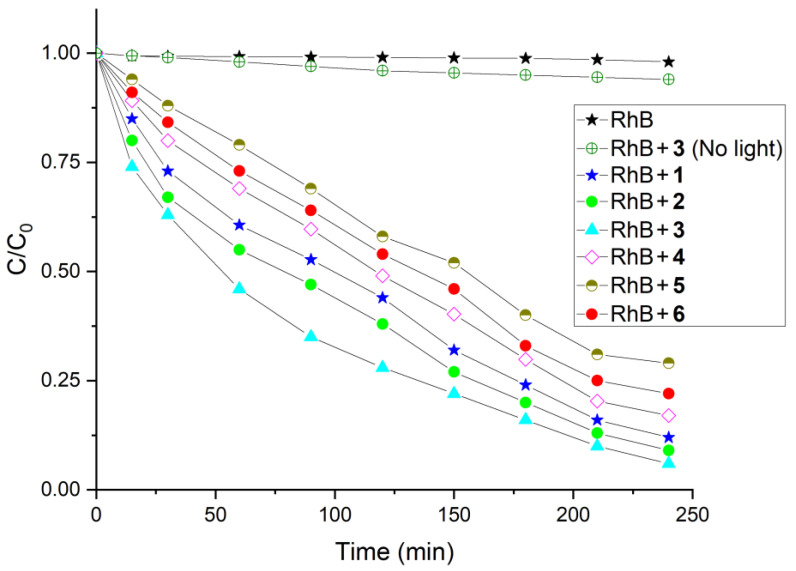
Photocatalytic degradation of RhB dye by six triads in aqueous solution (pH 7, temperature 298 K) under visible light irradiation.

Our planet suffers from pollution resulting from meeting the needs of industry. Textile, leather, drugs and paper printing processes discharge large quantities of dye into water ecosystems and damage the environment as a whole. Rhodamine B was one of the 20 dyes detected most frequently in wastewater, and it causes irritation to the skin, eyes, gastrointestinal tract, and respiratory system. As such, there is an urgent need to remove this dye from wastewater [45]. We have evaluated the photocatalytic activities of these nanostructured materials under visible light irradiation for their ability to degrade RhB dye in an aqueous solution. The minimal decay of RhB dye was observed in the absence of visible light or any of the nanomaterials **1**–**6**. Thus, visible light was determined to be essential to the photocatalytic properties of all these porphyrin nanoaggregates. Each experiment was performed with a 30 min delay after mixing the nanostructures and RhB in the dark to reach an adsorption–desorption equilibrium between the photocatalyst and RhB. Mass spectra were taken after 4 h for each experiment. New peaks (corresponding to some small molecules) in the mass spectra compared to RhB indicated that the RhB was successfully degraded in the presence of visible light irradiation and the photo-catalyst. The degradation process was monitored by UV–vis spectroscopy. The time-dependent absorption spectra of the aqueous RhB solution in the presence of nanostructures derived from triad **3** under visible light irradiation are shown in Figure S26. Decreasing the absorption peak intensity at 554 nm over time caused the photocatalytic degradation of RhB. Figure 4 shows the *C*/*C*_0_ versus time plot of RhB in the presence of various triad-based nanomaterials. A negligible degradation of the RhB dyes with the starting porphyrin monomer **SnP** or **ZnL^n^** was observed under the present experimental conditions.

Figure 4 shows that all the nanomaterials were active in the photodegradation of RhB in an aqueous solution. The degradation of RhB dye can be described by its degradation efficiency, (*C*_0_−*C*)/*C*_0_, where *C*_0_ is the initial concentration of RhB and *C* is the concentration at time *t*. The observed degradation rates of RhB are 89%, 91%, 94%, 84%, 72%, and 79% for the nanostructures derived from triads **1**, **2**, **3**, **4**, **5**, and **6**, respectively. The photocatalytic degradation efficiency of RhB dye in aqueous solution under visible light irradiation can be ranked as follows: **3** > **2** > **1** > **4** > **6** > **5**. Therefore, the nanoaggregates obtained from triad **3** show the best performance for the photocatalytic degradation of RhB. To further interpret the reaction kinetics involved in the decomposition of RhB dye, we used the pseudo-first-order model, as expressed by equation ln(*C*_0_/*C*) = *k*t, which is normally used for photocatalytic degradation experiments in which the initial concentration of pollutant or dye is low, where *k* is the pseudo-first-order rate constant. Based on the data plotted in Figure 4, Figure S27 represents the reaction kinetics of RhB decomposition. The first-order rate constant for the degradation of RhB by triad **3** (0.010 min^−1^) is higher than that for triad **2** (0.009 min^−1^), triad **1** (0.008 min^−1^), triad **4** (0.007 min^−1^), triad **6** (0.006 min^−1^), and triad **5** (0.005 min^−1^). These values are impressive compared to other values in the literature [45]. The recovery of these photocatalysts was very simple; we filtered the reaction mixture after the experiments and then washed it with water, followed by drying in air. Previously, we demonstrated that these catalysts were stable enough for further use (confirmed via the PXRD data after and before the reaction) [40]. Furthermore, the efficiency of the catalyst was not impaired, and showed excellent recyclability even after using it for the degradation of RhB in up to five cycles (Supplementary Figure S28).

A possible mechanism for the photocatalytic degradation of the RhB dye by porphyrin nanomaterials in an aqueous solution has previously been given [40,45]. When these nanomaterials are subjected to visible light irradiation along with RhB, the porphyrin nanomaterials absorb light, and the valence band (VB) electrons are elevated to the conduction band (CB) after crossing the bandgap. This causes the generation of electron–hole pairs (*e*^−^/*h*^+^) on the surfaces of the photo-catalysts. A strong intermolecular π electronic coupling occurs between the vicinal aggregations of these nanostructures. This can amplify the electronic delocalization occurring in the nanostructures that are held by strong intermolecular *π*–*π* interactions. The excited electrons in the conduction band may move without any constraint through the nanostructures. Therefore, the generated *e*^−^/*h*^+^ pairs were successfully isolated, and their recombination energy was significantly minimized. Hydroxyl radical (^•^OH) was produced by the reaction between photo-generated *e*^−^/*h*^+^ pairs and H_2_O or OH^−^ in the VB. On the other hand, the excited electrons in the CB interact with the dissolved oxygen in the water to form superoxide radical anions (O_2_^−^^•^). These photo-generated superoxide radical anions interact with H_2_O or OH^−^ to form hydroxyl radicals again. These highly active hydroxyl radical species oxidize the RhB molecules until they become degraded products on the surfaces of the photo-catalysts [46]. To detect the presence of hydroxyl radical during the photodegradation of RhB, we used coumarin as a radical scavenger. We repeated our degradation experiment with our catalyst in the presence of coumarin and irradiated the mixture with visible light for 30 min. After that, the fluorescences of the solutions excited at 325 nm were measured. We observed an intense peak at 460 nm compared to the coumarin (~400 nm). This result confirms that ^•^OH radicals were generated and reacted with coumarin to form a highly fluorescent 7-hydroxycoumarin (Supplementary Figures S29 and S30). In the presence of coumarin, the degradation efficiency of RhB drastically decreased. This suggests that a portion of the hydroxyl radicals was removed by coumarin to form 7-hydroxycoumarin, and the rest reacted with RhB until decomposition [47]. Similarly, for the detection of the superoxide radical anion, we used hydroethidine as a fluorescent probe. Upon reaction with the superoxide radical anion, hydroethidine (blue fluorescent) underwent oxygenation to form 2-hydroxyethidium (red fluorescent) (Figure S29) and showed a bright “turn-on” fluorescence at 610 nm [48]. A negative response to the ADPA (anthracene-9,10-dipropionic acid disodium salt) fluorescence test confirmed that singlet oxygen species were not generated during the degradation reaction [47].

## 3. Conclusions

In summary, six Zn(II)-Sn(IV)-Zn(II) porphyrin triads were synthesized via the complementary coordination approach. The cooperative metal–ligand coordination of the 3-pyridyl nitrogen in the Sn(IV)-porphyrin with the phenoxy Zn(II)-porphyrins, followed by the self-assembly process, determines the formation of these nanostructures. These triads formed different nanostructures due to the different substituents present on the axial Zn(II)-porphyrin moieties. Various types of interaction, including π–π stacking, Van der Waals forces, halogen–halogen interactions, and repulsions among electronegative atoms can contribute to the self-assembly of nanomaterials. These nanoaggregates also displayed efficient photocatalytic performances for the degradation of RhB dyes under visible light irradiation. The rate of the degradation of RhB by these nanomaterials in an aqueous solution varied from 72% to 95% within 4 h. The degree of self-assembly is mainly governed by the substituents on the triads, and reflects the photocatalytic performances. Our study suggests that the efficient photocatalytic performance of nanostructured porphyrin assemblies can be achieved by tuning the substituents of porphyrin molecules to generate specific morphologies. Furthermore, the easy synthesis, high efficiency, and high reproducibility of these nanomaterials based on complementary metalloporphyrins make them feasible for use in fabricating new porphyrin-based nanostructures for functional materials.

## 4. Materials and Methods

All the chemicals were procured from Sigma-Aldrich and used without further purification unless otherwise mentioned. *Trans*-dihydroxo-[5,15-bis(3-pyridyl)-10,20-bis(phenyl)porphyrinato]tin(IV) **SnP** was synthesized according to the previously reported procedure [40]. All the experiments reported here were carried out under inert argon atmosphere using standard Schlenk line techniques. The toluene was purified by distillation from a sodium/benzophenone ketyl solution, whereas pyrrole was derived from a solution over calcium hydride. Steady-state UV–vis spectra were recorded on a Shimadzu UV-3600 spectrophotometer (Shimadzu, Tokyo, Japan). The fluorescence spectra were recorded with a Shimadzu RF-5301PC fluorescence spectrophotometer (Shimadzu, Tokyo, Japan). The ^1^H-NMR spectra were obtained on a Bruker BIOSPIN/AVANCE III 400 spectrometer at 293 K (Bruker BioSpin GmbH, Silberstreifen, Rheinstetten, Germany). Electrospray ionization mass spectra (ESI-MS) were recorded on a Thermo Finnigan Linear Ion Trap Quadrupole mass spectrometer (Thermo Fisher Scientific, Waltham, MA, USA). Elemental analysis was performed using the EA 1110 Fisons analyzer (Used Lab Machines Limited, London, UK). Field emission scanning electron microscope (FE-SEM) images were obtained from MAIA III (TESCAN, Brno, Czech Republic).

### 4.1. General Procedure for the Synthesis of Free Porphyrin **H_2_L^n^**

Free porphyrin was synthesized by mixing aldehydes condensation in propionic acid using 1.0 equiv. of 4-hydroxybenzaldehyde, 3.0 equiv. of 4-substituted benzaldehyde, and 4.0 equiv. of pyrrole. In a typical reaction, pyrrole (0.77 mL, 11.1 mmol) was added dropwise to a solution of 4-substituted benzaldehyde (2.8 mmol) and benzaldehyde (0.89 g, 8.4 mmol) in propionic acid (250 mL) at reflux. After this the mixture was stirred for 1 h. The mixture was cooled to 0 °C to precipitate the product. The solid was filtered and washed with hot water, and then dried at 50 °C. Then, the mixture was purified by column chromatography (SiO_2_, eluent: CH_2_Cl_2_/MeOH) to afford the compound **H_2_L^n^**. The crude was then recrystallized from CH_2_Cl_2_/acetonitrile mixture to give a violet purple powder.

#### 4.1.1. Synthesis of *Meso*-5-(4-hydroxyphenyl)-10,15,20-tris(phenyl)porphyrin **H_2_L^1^**

A total of 0.89 g of benzaldehyde and eluent for column chromatography (CH_2_Cl_2_: MeOH = 98:2) was used. Yield: 0.123 g (7%). Analysis calculated for C_44_H_30_N_4_O: C, 83.79; H, 4.79; N, 8.88. Found: C, 84.02; H, 5.11; N, 8.80. ^1^H-NMR (400 MHz, CDCl_3_, ppm): δ –2.81 (s, 2H, NH), 7.14 (d, *J* = 8.4 Hz, 2H, H2,6-PhOH), 7.68–7.78 (m, 9H, H*m*,*p*-Ph), 8.11–8.19 (m, 8H, *meso*-O-Ar), 8.83 (s, 8H, β-pyrrole). UV–visible (CHCl_3_): *λ*_max_ (log *ε*), 418 (5.27), 517 (4.15), 554 (3.87), 593 (3.57), 649 (3.53) nm. Emission (CHCl_3_, *λ*_max_): 654, 713 nm.

#### 4.1.2. Synthesis of *Meso*-5-(4-hydroxyphenyl)-10,15,20-tris(4-methylphenyl)porphyrin **H_2_L^2^**

A total of 1.01 g of 4-methylbenzaldehyde and eluent for column chromatography (CH_2_Cl_2_: MeOH = 99:1) was used. Yield: 0.150 g (8%). Analysis calculated for C_47_H_36_N_4_O: C, 83.90; H, 5.39; N, 8.33. Found: C, 83.97; H, 5.57; N, 8.72. ^1^H-NMR (400 MHz, CDCl_3_, ppm): δ –2.79 (s, 2H, NH), 2.68 (s, 9H, Me), 7.15 (d, *J* = 8.4 Hz, 2H, H2,6-PhOH), 7.53 (d, *J* = 6.8 Hz, 6H, H3,5-Ar), 8.04–8.10 (m, 8H, *meso*-O-Ar), 8.83 (s, 8H, β-pyrrole). UV–visible (CHCl_3_): *λ*_max_ (log *ε*), 418 (5.25), 516 (4.14), 553 (3.86), 592 (3.56), 648 (3.52) nm. Emission (CHCl_3_, *λ*_max_): 654, 714 nm.

#### 4.1.3. Synthesis of *Meso*-5-(4-hydroxyphenyl)-10,15,20-tris(4-*tert*-butylphenyl)porphyrin **H_2_L^3^**

A total of 1.36 g of 4-*tert*-butylbenzaldehyde and eluent for column chromatography (CH_2_Cl_2_: MeOH = 99:1) was used. Yield: 0.112 g (5%). Analysis calculated for C_56_H_54_N_4_O: C, 84.17; H, 6.81; N, 7.01. Found: C, 84.02; H, 6.97; N, 6.88. ^1^H-NMR (400 MHz, CDCl_3_, ppm): δ –2.74 (s, 2H, NH), 1.58 (s, 27H, ^t^Bu), 7.17 (d, *J* = 8.7 Hz, 2H, H2,6-PhOH), 7.70 (d, *J* = 7.7 Hz, 6H, H3,5-Ar), 8.08–8.16 (m, 8H, *meso*-O-Ar), 8.87 (d, *J* = 4.4 Hz, 8H, β-pyrrole). UV–visible (CHCl_3_): *λ*_max_ (log *ε*), 419 (5.24), 517 (4.15), 554 (3.84), 592 (3.57), 648 (3.55) nm. Emission (CHCl_3_, *λ*_max_): 653, 715 nm.

#### 4.1.4. Synthesis of *Meso*-5-(4-hydroxyphenyl)-10,15,20-tris(4-methoxyphenyl)porphyrin **H_2_L^4^**

A total of 1.14 g of 4-methoxybenzaldehyde and eluent for column chromatography (CH_2_Cl_2_: MeOH = 96:4) was used. Yield: 0.140 g (7%). Analysis calculated for C_47_H_36_N_4_O_4_: C, 78.31; H, 5.03; N, 7.77. Found: C, 78.22; H, 5.41; N, 7.69. ^1^H-NMR (400 MHz, CDCl_3_, ppm): δ –2.75 (s, 2H, NH), 4.08 (s, 9H, OMe), 7.16 (d, *J* = 8.6 Hz, 2H, H2,6-PhOH), 7.36 (d, *J* = 7.6 Hz, 6H, H3,5-Ar), 8.08–8.14 (m, 8H, *meso*-O-Ar), 8.94 (s, 8H, β-pyrrole). UV–visible (CHCl_3_): *λ*_max_ (log *ε*), 420 (5.27), 519 (4.16), 554 (3.86), 593 (3.56), 649 (3.54) nm. Emission (CHCl_3_, *λ*_max_): 655, 719 nm.

#### 4.1.5. Synthesis of *Meso*-5-(4-hydroxyphenyl)-10,15,20-tris(4-chlorophenyl)porphyrin **H_2_L^5^**

A total of 1.18 g of 4-chlorobenzaldehyde and eluent for column chromatography (CH_2_Cl_2_: MeOH = 97:3) was used. Yield: 0.141 g (6%). Analysis calculated for C_44_H_27_N_4_Cl_3_O: C, 71.99; H, 3.71; N, 7.63. Found: C, 71.87; H, 3.95; N, 7.57. ^1^H-NMR (400 MHz, CDCl_3_, ppm): δ –2.86 (s, 2H, NH), 7.12 (d, *J* = 7.2 Hz, 2H, H2,6-PhOH), 7.72 (d, *J* = 7.6 Hz, 6H, H3,5-Ar), 8.04–8.08 (m, *meso*-O-Ar), 8.84 (d, *J* = 4.0 Hz, 8H, β-pyrrole). UV–visible (CHCl_3_): *λ*_max_ (log *ε*), 418 (5.29), 516 (4.17), 552 (3.89), 591 (3.59), 649 (3.56) nm. Emission (CHCl_3_, *λ*_max_): 654, 714 nm.

#### 4.1.6. Synthesis of *Meso*-5-(4-hydroxyphenyl)-10,15,20-tris(4-nitrophenyl)porphyrin **H_2_L^6^**

A total of 1.27 g of 4-nitrobenzaldehyde and eluent for column chromatography (CH_2_Cl_2_: MeOH = 95:5) was used. Yield: 0.128 g (6%). Analysis calculated for C_44_H_27_N_7_O_7_: C, 69.02; H, 3.55; N, 12.80. Found: C, 69.23; H, 3.87; N, 12.73. ^1^H-NMR (400 MHz, CDCl_3_, ppm): δ –2.94 (s, 2H, NH), 7.22 (d, *J* = 8.6 Hz, 2H, H2,6-PhOH), 8.16–8.31 (m, 8H, H3,5-Ar + H3,5-PhOH), 8.65 (d, *J* = 7.8 Hz, 6H, *meso*-O-Ar), 8.90 (s, 8H, β-pyrrole). UV–visible (CHCl_3_): *λ*_max_ (log *ε*), 420 (5.26), 519 (4.16), 553 (3.87), 592 (3.58), 648 (3.55) nm. Emission (CHCl_3_, *λ*_max_): 653, 719 nm.

### 4.2. General Procedure for the Synthesis of Zn(II)-Porphyrin **ZnL^n^**

In each case, Zn(OAc)_2_∙2H_2_O (0.053 g, 0.29 mmol) was added to a solution of **H_2_L^n^** (0.12 mmol) in a mixed solvent of tetrahydrofuran (20 mL) and methanol (20 mL). The mixture was refluxed for 2 h. The solvent was then removed, and the solid product was purified by column chromatography (SiO_2_, eluent: CH_2_Cl_2/_MeOH). The crude was recrystallized from CH_2_Cl_2_/hexane to give a brownish red.

#### 4.2.1. Synthesis of *Meso*-5-(4-hydroxyphenyl)-10,15,20-tris(phenyl)porphyrinato)zinc(II) **ZnL^1^**

A total of 0.076 g of **H_2_L^1^** and eluent for column chromatography (CH_2_Cl_2_: MeOH = 98:2) was used. Yield: 0.075 g (90%). Analysis calculated for C_68_H_76_N_4_OZn: C, 76.14; H, 4.07; N, 8.07. Found: C, 76.01; H, 4.39; N, 7.93. ^1^H-NMR (400 MHz, DMSO-d_6_, ppm): δ 7.17 (d, *J* = 8.6 Hz, 2H, H2,6-PhOH), 7.76–7.82 (m, 9H, H*m*,*p*-Ph), 7.97 (d, *J* = 8.8 Hz, 2H, H3,5-PhOH), 8.15–8.21 (m, 6H, *meso*-O-Ar), 8.77 (d, *J* = 4.0 Hz, 6H, β-pyrrole), 8.86 (d, *J* = 3.6 Hz, 2H, β-pyrrole), 9.87 (s, 1H, OH). UV–visible (CHCl_3_): *λ*_max_ (log *ε*), 421 (5.31), 547 (4.05), 585 (3.71) nm. Emission (CHCl_3_, *λ*_max_): 595, 642 nm.

#### 4.2.2. Synthesis of *Meso*-5-(4-hydroxyphenyl)-10,15,20-tris(4-methylphenyl)porphyrinato)zinc(II) **ZnL^2^**

A total of 0.081 g of **H_2_L^2^** and eluent for column chromatography (CH_2_Cl_2_: MeOH = 99:1) was used. Yield: 0.084 g (95%). Analysis calculated for C_68_H_76_N_4_OZn: C, 76.68; H, 4.66; N, 7.61. Found: C, 76.54; H, 4.79; N, 7.63. ^1^H-NMR (400 MHz, DMSO-d_6_, ppm): δ 2.67 (s, 9H, Me), 7.17 (d, *J* = 7.6 Hz, 2H, H2,6-PhOH), 7.58 (d, *J* = 6.8 Hz, 6H, H3,5-Ar), 7.95 (d, *J* = 7.6 Hz, 2H, H3,5-PhOH), 8.04 (d, *J* = 6.8 Hz, 6H, *meso*-O-Ar), 8.77 (d, *J* = 4.0 Hz, 6H, β-pyrrole), 8.83 (d, *J* = 3.6 Hz, 2H, β-pyrrole), 9.85 (s, 1H, OH). UV–visible (CHCl_3_): *λ*_max_ (log *ε*), 421 (5.29), 548 (4.04), 586 (3.70) nm. Emission (CHCl_3_, *λ*_max_): 597, 643 nm.

#### 4.2.3. Synthesis of *Meso*-5-(4-hydroxyphenyl)-10,15,20-tris(4-*tert*-butylphenyl)porphyrinato)zinc(II) **ZnL^3^**

A total of 0.096 g of **H_2_L^3^** and eluent for column chromatography (CH_2_Cl_2_: MeOH = 99:1) was used. Yield: 0.097 g (94%). Analysis calculated for C_68_H_76_N_4_OZn: C, 77.99; H, 6.08; N, 6.50. Found: C, 77.65; H, 6.22; N, 6.45. ^1^H-NMR (400 MHz, DMSO-d_6_, ppm): δ 1.58 (s, 27H, ^t^Bu), 7.17 (d, *J* = 8.8 Hz, 2H, H2,6-PhOH), 7.80 (d, *J* = 8.0 Hz, 6H, H3,5-Ar), 7.95 (d, *J* = 7.6 Hz, 2H, H3,5-PhOH), 8.10 (d, *J* = 7.2 Hz, 6H, *meso*-O-Ar), 8.74–8.80 (m, 6H, β-pyrrole), 8.83 (d, *J* = 4.4 Hz, 2H, β-pyrrole), 9.86 (s, 1H, OH). UV–visible (CHCl_3_): *λ*_max_ (log *ε*), 421 (5.27), 549 (4.03), 586 (3.69) nm. Emission (CHCl_3_, *λ*_max_): 597, 644 nm.

#### 4.2.4. Synthesis of *Meso*-5-(4-hydroxyphenyl)-10,15,20-tris(4-methoxyphenyl)porphyrinato)zinc(II) **ZnL^4^**

A total of 0.086 g of **H_2_L^4^** and eluent for column chromatography (CH_2_Cl_2_: MeOH = 97:3) was used. Yield: 0.090 g (96%). Analysis calculated for C_68_H_76_N_4_OZn: C, 71.99; H, 4.37; N, 7.14. Found: C, 71.74; H, 4.58; N, 7.07. ^1^H-NMR (400 MHz, DMSO-d_6_, ppm): δ 4.05 (s, 9H, OMe), 7.17 (d, *J* = 8.4 Hz, 2H, H2,6-PhOH), 7.34 (d, *J* = 8.5 Hz, 6H, H3,5-Ar), 7.96 (d, *J* = 8.2 Hz, 2H, H3,5-PhOH), 8.08 (d, *J* = 8.5 Hz, 6H, *meso*-O-Ar), 8.79 (d, *J* = 4.0 Hz, 6H, β-pyrrole), 8.82 (d, *J* = 3.8 Hz, 2H, β-pyrrole), 9.85 (s, 1H, OH). UV–visible (CHCl_3_): *λ*_max_ (log *ε*), 422 (5.25), 550 (4.02), 588 (3.68) nm. Emission (CHCl_3_, *λ*_max_): 599, 645 nm.

#### 4.2.5. Synthesis of *Meso*-5-(4-hydroxyphenyl)-10,15,20-tris(4-chlorophenyl)porphyrinato)zinc(II) **ZnL^5^**

A total of 0.088 g of **H_2_L^5^** and eluent for column chromatography (CH_2_Cl_2_: MeOH = 97:3) was used. Yield: 0.088 g (92%). Analysis calculated for C_68_H_76_N_4_OZn: C, 66.27; H, 3.16; N, 7.03. Found: C, 66.03; H, 3.27; N, 6.95. ^1^H-NMR (400 MHz, DMSO-d_6_, ppm): δ 7.18 (d, *J* = 8.4 Hz, 2H, H2,6-PhOH), 7.85 (d, *J* = 7.6 Hz, 6H, H3,5-Ar), 7.96 (d, *J* = 8.4 Hz, 2H, H3,5-PhOH), 8.17 (d, *J* = 7.2 Hz, 6H, *meso*-O-Ar), 8.78 (d, *J* = 6.0 Hz, 6H, β-pyrrole), 8.87 (d, *J* = 4.4 Hz, 2H, β-pyrrole), 9.89 (s, 1H, OH). UV–visible (CHCl_3_): *λ*_max_ (log *ε*), 420 (5.23), 547 (4.03), 585 (3.70) nm. Emission (CHCl_3_, *λ*_max_): 598, 642 nm.

#### 4.2.6. Synthesis of *Meso*-5-(4-hydroxyphenyl)-10,15,20-tris(4-nitrophenyl)porphyrinato)zinc(II) **ZnL^6^**

A total of 0.092 g of **H_2_L^6^** and eluent for column chromatography (CH_2_Cl_2_: MeOH = 95:5) was used. Yield: 0.089 g (90%). Analysis calculated for C_44_H_25_N_7_O_7_Zn: C, 63.74; H, 3.04; N, 11.83. Found: C, 63.44; H, 3.42; N, 11.65. ^1^H-NMR (400 MHz, DMSO-d_6_, ppm): δ 7.23 (d, *J* = 8.5 Hz, 2H, H2,6-PhOH), 8.20 (d, *J* = 8.2 Hz, 2H, H3,5-PhOH), 8.34 (d, *J* = 7.6 Hz, 6H, H3,5-Ar), 8.66 (d, *J* = 7.8 Hz, 6H, *meso*-O-Ar), 8.87 (d, *J* = 4.2 Hz, 6H, β-pyrrole), 8.92 (d, *J* = 3.8 Hz, 2H, β-pyrrole), 9.84 (s, 1H, OH). UV–visible (CHCl_3_): *λ*_max_ (log *ε*), 419 (5.25), 548 (4.04), 586 (3.67) nm. Emission (CHCl_3_, *λ*_max_): 600, 644 nm.

### 4.3. General Procedure for the Synthesis of the Triad

A mixture of **ZnL^n^** (0.08 mmol) and **SnP** (0.031 g, 0.04 mmol) was added to anhydrous toluene (20 mL) and refluxed for 48 h under an argon atmosphere. Then, the reaction mixture was cooled to room temperature, and we filtered the colored precipitate. After that, it was washed with toluene, then dried under a vacuum.

#### 4.3.1. Synthesis of Triad **1**

A total of 0.056 g of **ZnL^1^** was used, and light green precipitate was obtained. Yield: (0.068 g, 80%). Analysis calculated for C_130_H_80_N_14_O_2_SnZn_2_: C, 73.66; H, 3.80; N, 9.25. Found: C, 73.34; H, 4.12; N, 9.14. ^1^H-NMR (400 MHz, DMSO-d_6_, ppm): δ 2.66 (d, *J* = 8.6 Hz, 4H, α-bridging phenyl), 6.70 (d, *J* = 8.8 Hz, 4H, β-bridging phenyl), 7.19–7.31 (m, 18H, {*m*,*p*-phenyl}-axial), 7.52–7.62 (m, 20H, *meso*-O-Phenyl-axial + H5-Py-central + *m*,*p*-Phenyl-central), 8.07–8.11 (m, 6H, H4-Py-central + *meso*-O-phenyl-central), 8.40 (d, *J* = 7.8 Hz, 4H, β-pyrrole-axial), 8.55 (d, *J* = 7.8 Hz, 4H, β-pyrrole-axial), 8.65–8.75 (m, 8H, β-pyrrole-axial), 9.05 (d, *J* = 8.2 Hz, 4H, β-pyrrole-central), 9.15 (d, *J* = 8.2 Hz, 4H, β-pyrrole-central), 9.28 (d, *J* = 5.0 Hz, 2H, H6-Py), 9.58 (s, 2H, H2-Py). UV–visible (CHCl_3_): *λ*_max_ (log *ε*), 454 (5.65), 573 (5.21), 615 (5.19) nm. Emission (CHCl_3_, *λ*_max_): 592, 642 nm.

#### 4.3.2. Synthesis of Triad **2**

A total of 0.059 g of **ZnL^2^** was used, and light green precipitate was obtained. Yield: (0.074 g, 84%). Analysis calculated for C_136_H_92_N_14_O_2_SnZn_2_: C, 74.12; H, 4.21; N, 8.90. Found: C, 73.94; H, 4.32; N, 8.74. ^1^H-NMR (400 MHz, DMSO-d_6_, ppm): δ 2.64 (d, *J* = 8.6 Hz, 4H, α-bridging phenyl), 2.69 (s, 18H, Me), 6.70 (d, *J* = 8.8 Hz, 4H, β-bridging phenyl), 7.24–7.32 (m, 12H, *m*-Ar-axial), 7.52–7.60 (m, 8H, H5-Py-central + *m*,*p*-Phenyl-central), 7.68–7.74 (m, 12H, *meso*-O-Ar-axial), 8.08–8.14 (m, 6H, H4-Py-central + *meso*-O-phenyl-central), 8.42 (d, *J* = 7.8 Hz, 4H, β-pyrrole-axial), 8.57 (d, *J* = 7.8 Hz, 4H, β-pyrrole-axial), 8.70–8.80 (m, 8H, β-pyrrole-axial), 9.08 (d, *J* = 8.2 Hz, 4H, β-pyrrole-central), 9.15 (d, *J* = 8.2 Hz, 4H, β-pyrrole-central), 9.26 (d, *J* = 5.0 Hz, 2H, H6-Py), 9.54 (s, 2H, H2-Py). UV–visible (CHCl_3_): *λ*_max_ (log *ε*), 458 (5.88), 577 (5.68), 618 (5.69) nm. Emission (CHCl_3_, *λ*_max_): 592, 642 nm.

#### 4.3.3. Synthesis of Triad **3**

A total of 0.069 g of **ZnL^3^** was used, and green precipitate was obtained. Yield: (0.082 g, 84%). Analysis calculated for C_154_H_128_N_14_O_2_SnZn_2_: C, 75.30; H, 5.25; N, 7.98. Found: C, 75.02; H, 5.32; N, 7.87. ^1^H-NMR (400 MHz, DMSO-d_6_, ppm): δ 1.58 (s, 54H, ^t^Bu), 2.62 (d, *J* = 8.6 Hz, 4H, α-bridging phenyl), 6.72 (d, *J* = 8.6 Hz, 4H, β-bridging phenyl), 7.42–7.52 (m, 20H, *m*,*p*-phenyl-central + H5-Py-central + *m*-Ar-axial), 7.65–7.73 (m, 12H, *meso*-O-Ar-axial), 8.08–8.16 (m, 6H, H4-Py-central + *meso*-O-phenyl-central), 8.44 (d, *J* = 7.8 Hz, 4H, β-pyrrole-axial), 8.56 (d, *J* = 7.8 Hz, 4H, β-pyrrole-axial), 8.68–8.76 (m, 8H, β-pyrrole-axial), 9.08 (d, *J* = 8.2 Hz, 4H, β-pyrrole-central), 9.17 (d, *J* = 8.2 Hz, 4H, β-pyrrole-central), 9.30 (d, *J* = 5.0 Hz, 2H, H6-Py), 9.55 (s, 2H, H2-Py). UV–visible (CHCl_3_): *λ*_max_ (log *ε*), 454 (5.88), 577 (5.68), 619 (5.70) nm. Emission (CHCl_3_, *λ*_max_): 591, 640 nm.

#### 4.3.4. Synthesis of Triad **4**

A total of 0.063 g of **ZnL^4^** was used, and dark blue precipitate was obtained. Yield: (0.083 g, 90%). Analysis calculated for C_136_H_92_N_14_O_8_SnZn_2_: C, 71.03; H, 4.03; N, 8.53. Found: C, 70.91; H, 4.30; N, 8.44. ^1^H-NMR (400 MHz, DMSO-d_6_, ppm): δ 2.70 (d, *J* = 8.6 Hz, 4H, α-bridging phenyl), 4.08 (s, 18H, OMe), 6.73 (d, *J* = 8.6 Hz, 4H, β-bridging phenyl), 7.22–7.32 (m, 12H, *m*-phenyl), 7.35–7.43 (m, 8H, H5-Py-central + *m*,*p*-phenyl-central), 7.48–7.58 (m, 12H, *meso*-O-Ar-axial), 8.06–8.12 (m, 6H, H4-Py-central + *meso*-O-phenyl-central), 8.38 (d, *J* = 7.8 Hz, 4H, β-pyrrole-axial), 8.55 (d, *J* = 7.8 Hz, 4H, β-pyrrole-axial), 8.65–8.75 (m, 8H, β-pyrrole-axial), 9.07 (d, *J* = 8.2 Hz, 4H, β-pyrrole-central), 9.14 (d, *J* = 8.2 Hz, 4H, β-pyrrole-central), 9.28 (d, *J* = 5.0 Hz, 2H, H6-Py), 9.57 (s, 2H, H2-Py). UV–visible (CHCl_3_): *λ*_max_ (log *ε*), 423 (5.71), 458 (5.32), 574 (5.12), 616 (5.09) nm. Emission (CHCl_3_, *λ*_max_): 596, 644 nm.

#### 4.3.5. Synthesis of Triad **5**

A total of 0.064 g of **ZnL^5^** was used, and blue precipitate was obtained. Yield: (0.076 g, 82%). Analysis calculated for C_130_H_74_Cl_6_N_14_O_2_SnZn_2_: C, 67.12; H, 3.21; N, 8.43. Found: C, 66.97; H, 3.55; N, 8.20. ^1^H-NMR (400 MHz, DMSO-d_6_, ppm): δ 2.65 (d, *J* = 8.6 Hz, 4H, α-bridging phenyl), 6.74 (d, *J* = 8.6 Hz, 4H, β-bridging phenyl), 7.22–7.30 (m, 8H, *m*,*p*-phenyl-central + H5-Py-central), 7.42–7.52 (m, 12H, *m*-Ar-axial), 7.62–7.70 (m, 12H, *meso*-O-Ar-axial), 8.06–8.12 (m, 6H, H4-Py-central + *meso*-O-phenyl-central), 8.36 (d, *J* = 7.8 Hz, 4H, β-pyrrole-axial), 8.48 (d, *J* = 7.8 Hz, 4H, β-pyrrole-axial), 8.60–8.70 (m, 8H, β-pyrrole-axial), 9.05 (d, *J* = 8.2 Hz, 4H, β-pyrrole-central), 9.12 (d, *J* = 8.2 Hz, 4H, β-pyrrole-central), 9.28 (d, *J* = 5.0 Hz, 2H, H6-Py), 9.52 (s, 2H, H2-Py). UV–visible (CHCl_3_): *λ*_max_ (log *ε*), 422 (5.85), 458 (5.69), 498 (5.69), 582 (5.61), 623 (5.65) nm. Emission (CHCl_3_, *λ*_max_): 595, 645 nm.

#### 4.3.6. Synthesis of Triad **6**

A total of 0.066 g of **ZnL^6^** was used, and brown precipitate was obtained. Yield: (0.082 g, 86%). Analysis calculated for C_130_H_74_N_20_O_14_SnZn_2_: C, 65.34; H, 3.12; N, 11.72. Found: C, 65.11; H, 3.40; N, 11.60. ^1^H-NMR (400 MHz, DMSO-d_6_, ppm): δ 2.74 (d, *J* = 8.6 Hz, 4H, α-bridging phenyl), 6.80 (d, *J* = 8.6 Hz, 4H, β-bridging phenyl), 7.22–7.31 (m, 8H, *m*,*p*-phenyl-central + H5-Py-central), 8.04–8.12 (m, 6H, H4-Py-central + *meso*-O-phenyl-axial), 8.15–8.16 (m, 12H, *m*-Ar-axial), 8.30–8.40 (m, 12H, *meso*-O-Ar-axial), 8.48 (d, *J* = 7.8 Hz, 4H, β-pyrrole-axial), 8.62 (d, *J* = 7.8 Hz, 4H, β-pyrrole-axial), 8.70–8.80 (m, 8H, β-pyrrole-axial), 9.06 (d, *J* = 8.2 Hz, 4H, β-pyrrole-central), 9.16 (d, *J* = 8.2 Hz, 4H, β-pyrrole-central), 9.26 (d, *J* = 5.0 Hz, 2H, H6-Py), 9.52 (s, 2H, H2-Py). UV–visible (CHCl_3_): *λ*_max_ (log *ε*), 424 (5.96), 455 (5.21), 572 (5.07), 618 (5.04) nm. Emission (CHCl_3_, *λ*_max_): 597, 647 nm.

### 4.4. Photocatalytic Degradation of Rhodamine B by Nanoaggregates of Triad **1**–**6**

In the typical process, 10 mg of the photocatalyst was added to 100 mL of aqueous RhB solution (4.5 mg L^−1^, pH 7) under stirring at room temperature. Before the visible light irradiation, the mixture was kept in the dark for 30 min in order to reach an adsorption–desorption equilibrium. After that, the mixture was exposed to visible light irradiation from a 150 W Xenon arc lamp (ABET technologies, Milford, CT, USA) at room temperature. All the samples were collected by centrifugation at given time intervals to determine the RhB concentration via UV–vis spectroscopy at 554 nm.

## Data Availability

Data are available in the article and supplementary materials.

## Supplementary Materials

^1^H-NMR spectra, mass spectra, FE-SEM images, and the photocatalytic degradation of RhB are included in Figures S1–S30. Figure S1: 1H-NMR spectrum of H2L1, Figure S2: 1H-NMR spectrum of H2L2, Figure S3: 1H-NMR spectrum of H2L3, Figure S4: 1H-NMR spectrum of H2L4, Figure S5: 1H-NMR spectrum of H2L5, Figure S6: 1H-NMR spectrum of H2L6, Figure S7: 1H-NMR spectrum of ZnL1, Figure S8: 1H-NMR spectrum of ZnL2, Figure S9: 1H-NMR spectrum of ZnL3, Figure S10: 1H-NMR spectrum of ZnL4, Figure S11: 1H-NMR spectrum of ZnL5, Figure S12: 1H-NMR spectrum of ZnL6, Figure S13: 1H-NMR spectrum of 1, Figure S14: 1H-NMR spectrum of 2, Figure S15: 1H-NMR spectrum of 3, Figure S16: 1H-NMR spectrum of 4, Figure S17: 1H-NMR spectrum of 5, Figure S18: 1H-NMR spectrum of 6, Figure S19: ESI-MS spectrum of 1, Figure S20: ESI-MS spectrum of 2, Figure S21: ESI-MS spectrum of 3, Figure S22: ESI-MS spectrum of 4, Figure S23: ESI-MS spectrum of 5, Figure S24: ESI-MS spectrum of 6, Figure S25: Lower magnification of FE-SEM images for the assembly patterns of triads (1–6), Figure S26: Absorption spectra of RhB in the presence of nanostructures derived from triad 3 under visible light irradiation, Figure S27: Kinetics for the photocatalytic degradation of RhB under visible light irradiation of the six triads (1–6), Figure S28: Catalytic cycles (up to 5 cycles) using triad 3 as a photocatalyst for the degradation of RhB, Figure S29: Schematic representation of the detection of the hydroxyl and superoxide radical anion that were generated during the photodegradation experiments, Figure S30: UV–visible spectra of coumarin in the presence of triad 3 in water. λe_x_ = 325 nm and light exposure time (30 min).

## Sample Availability

Samples of the compounds are not available from the authors.
