# Supplementary material for: Self-Assembled Nanomaterials Based on Complementary Sn(IV) and Zn(II)-Porphyrins, and Their Photocatalytic Degradation for Rhodamine B Dye
Source: Molecules. 2021 Jun 11;26(12):3598. doi: 10.3390/molecules26123598 (PMC8231224; doi:10.3390/molecules26123598)
Supplement: Supplementary file 1 [file molecules-26-03598-s001.zip › molecules-1224198-supplementary.pdf]

Supplementary Materials

Self-assembled Nanomaterials Based on Complementary Sn(IV) and Zn(II)-porphyrins, and Their Photocatalytic Degradation for Rhodamine B Dye

Nirmal Kumar Shee and Hee-Joon Kim*

Department of Applied Chemistry, Kumoh National Institute of Technology
61 Daehak-ro, Gumi 39177, Republic of Korea

List of contents:

Figure S1. ^{1}H NMR spectrum of *meso*-5-(4-hydroxyphenyl)-10,15,20-tris(phenyl)porphyrin $\mathbf{H_2L^1}$ in $CDCl_3$.

Figure S2. ^{1}H NMR spectrum of *meso*-5-(4-hydroxyphenyl)-10,15,20-tris(4-methyl-phenyl)porphyrin $\mathbf{H_2L^2}$ in $CDCl_3$.

Figure S3. ^{1}H NMR spectrum of *meso*-5-(4-hydroxyphenyl)-10,15,20-tris(4-*tert*-butylphenyl)porphyrin $\mathbf{H_2L^3}$ in $CDCl_3$.

Figure S4. ^{1}H NMR spectrum of *meso*-5-(4-hydroxyphenyl)-10,15,20-tris(4-methoxy-phenyl)porphyrin $\mathbf{H_2L^4}$ in $CDCl_3$.

Figure S5. ^{1}H NMR spectrum of *meso*-5-(4-hydroxyphenyl)-10,15,20-tris(4-chloro-phenyl)porphyrin $\mathbf{H_2L^5}$ in $CDCl_3$.

Figure S6. ^{1}H NMR spectrum of *meso*-5-(4-hydroxyphenyl)-10,15,20-tris(4-nitro-phenyl)porphyrin $\mathbf{H_2L^6}$ in $CDCl_3$.

Figure S7. ^{1}H NMR spectrum of *meso*-5-(4-hydroxyphenyl)-10,15,20-tris(phenyl)porphyrinato)zinc(II) **ZnL^1** in DMSO-d_6.

Figure S8. ^{1}H NMR spectrum of *meso*-5-(4-hydroxyphenyl)-10,15,20-tris(4-methyl-phenyl)porphyrinato)zinc(II) **ZnL^2** in DMSO-d_6.

Figure S9. ^{1}H NMR spectrum of *meso*-5-(4-hydroxyphenyl)-10,15,20-tris(4-*tert*-butyl-phenyl)porphyrinato)zinc(II) **ZnL^3** in DMSO-d_6.

Figure S10. ^{1}H NMR spectrum of *meso*-5-(4-hydroxyphenyl)-10,15,20-tris(4-methoxy-phenyl)porphyrinato)zinc(II) **ZnL^4** in DMSO-d_6.

Figure S11. ^{1}H NMR spectrum of *meso*-5-(4-hydroxyphenyl)-10,15,20-tris(4-chloro-phenyl)porphyrinato)zinc(II) **ZnL^5** in DMSO-d_6.

Figure S12. ^{1}H NMR spectrum of *meso*-5-(4-hydroxyphenyl)-10,15,20-tris(4-nitrophenyl)porphyrinato)zinc(II) **ZnL^6** in DMSO-d_6.

Figure S13. ^{1}H NMR spectrum of triad **1** in DMSO-d_6.

Figure S14. ^{1}H NMR spectrum of triad **2** in DMSO-d_6.

Figure S15. ^{1}H NMR spectrum of triad **3** in DMSO-d_6.

Figure S16. ^{1}H NMR spectrum of triad **4** in DMSO-d_6.

Figure S17. ^{1}H NMR spectrum of triad **5** in DMSO-d_6.

Figure S18. ^{1}H NMR spectrum of triad **6** in DMSO-d_6.

Figure S19. ESI-MS spectrum of triad **1**.

Figure S20. ESI-MS spectrum of triad **2**.

Figure S21. ESI-MS spectrum of triad **3**.

Figure S22. ESI-MS spectrum of triad **4**.

Figure S23. ESI-MS spectrum of triad **5**.

Figure S24. ESI-MS spectrum of triad **6**.

Figure S25. Lower magnification of FE-SEM images for the assembly patterns of triads: (a) **1**; (b) **2**; (c) **3**; (d) **4**; (e) **5**; (f) **6**.

Figure S26. Absorption spectra of RhB in the presence of nano fibers derived from triad **3** under visible light irradiation.

Figure S27. Kinetics for the photocatalytic degradation of RhB under visible light irradiation of the six triads (**1-6**).

Figure S28. Catalytic cycles (up to 5 cycles) using triad **3** as a photocatalyst for the degradation of RhB.

Figure S29. Schematic representation of the detection of the hydroxyl and superoxide radical anion that were generated during the photodegradation experiments.

Figure S30. UV-visible spectra of coumarin in the presence of triad **3** in water. λ_{ex} = 325 nm and light exposure time (30 min).

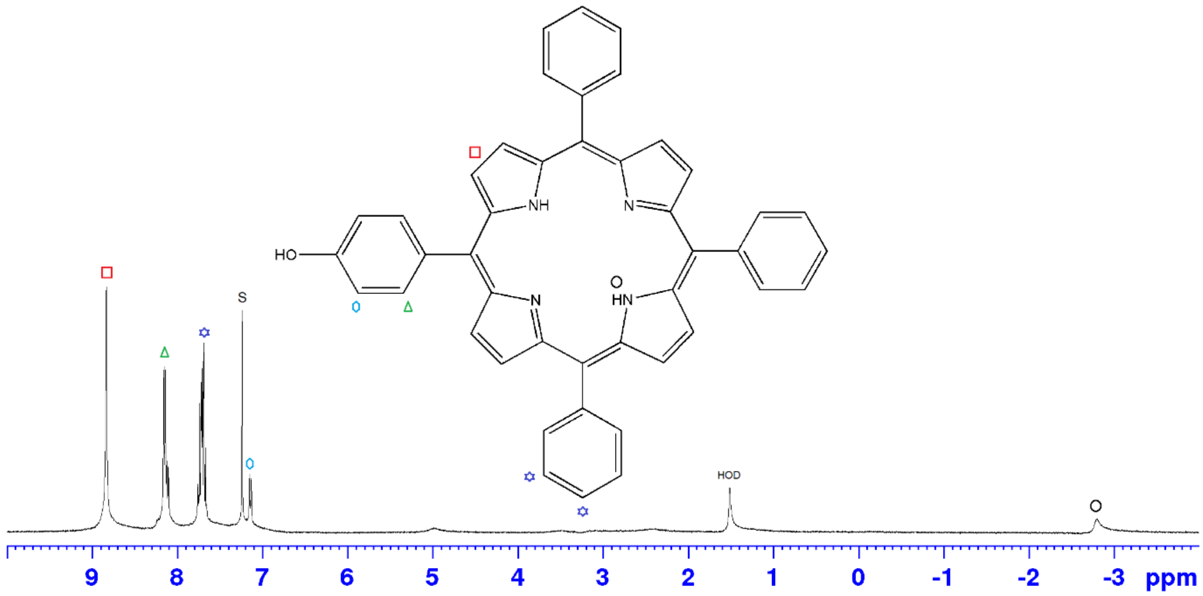

Figure S1. ^{1}H NMR spectrum of *meso*-5-(4-hydroxyphenyl)-10,15,20-tris(phenyl)porphyrin $\mathbf{H_2L^1}$ in $CDCl_3$.

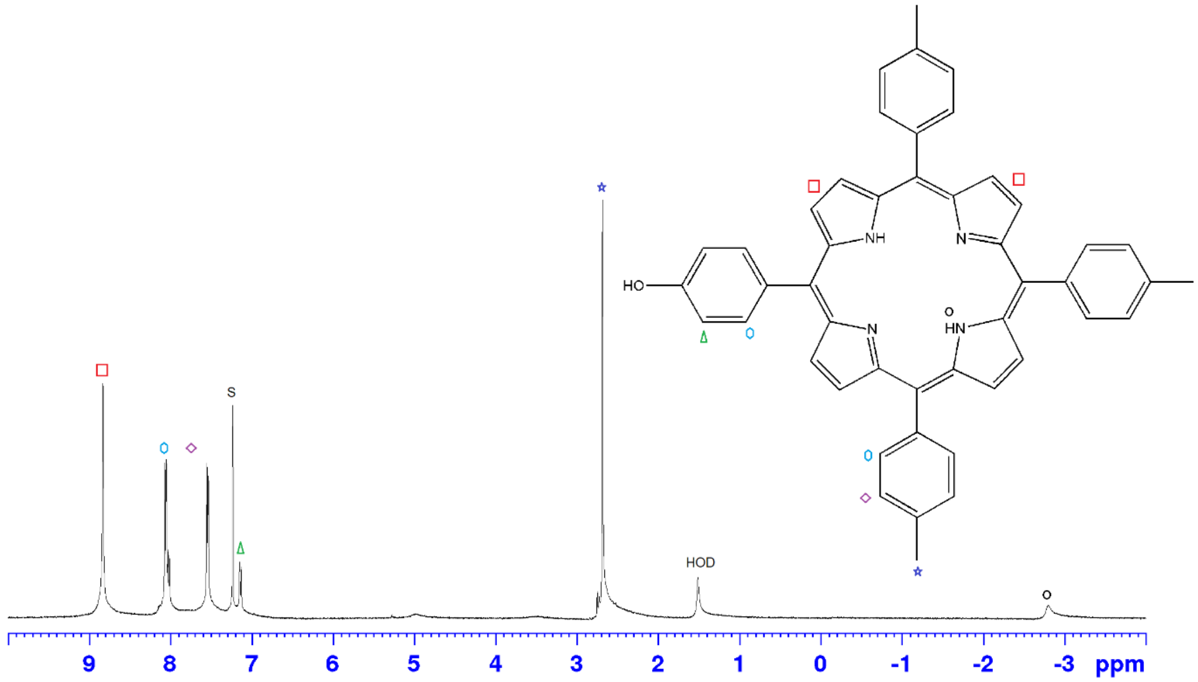

Figure S2. ^{1}H NMR spectrum of *meso*-5-(4-hydroxyphenyl)-10,15,20-tris(4-methyl-phenyl)porphyrin $\mathbf{H_2L^2}$ in $CDCl_3$.

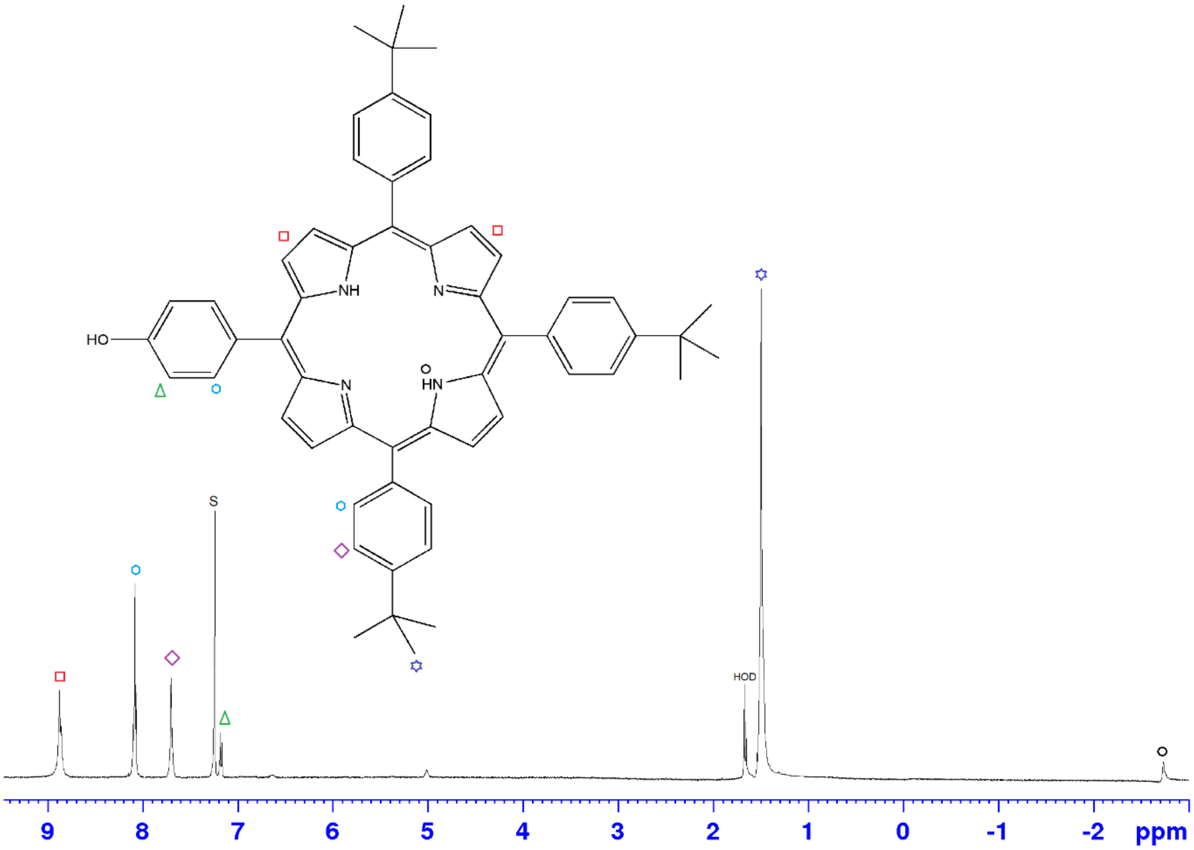

Figure S3. ^{1}H NMR spectrum of *meso*-5-(4-hydroxyphenyl)-10,15,20-tris(4-*tert*-butyl-phenyl)porphyrin $\mathbf{H_2L^3}$ in $CDCl_3$.

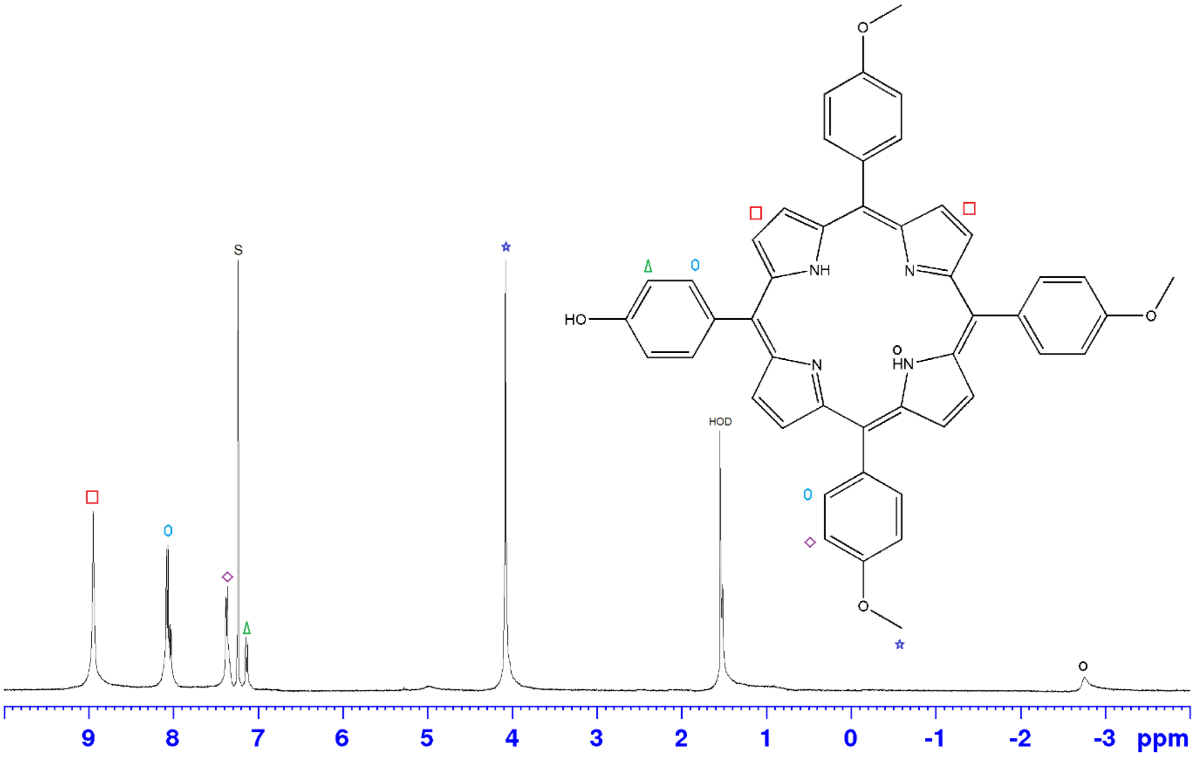

Figure S4. ^{1}H NMR spectrum of *meso*-5-(4-hydroxyphenyl)-10,15,20-tris(4-methoxy-phenyl)porphyrin $\mathbf{H_2L^4}$ in $CDCl_3$.

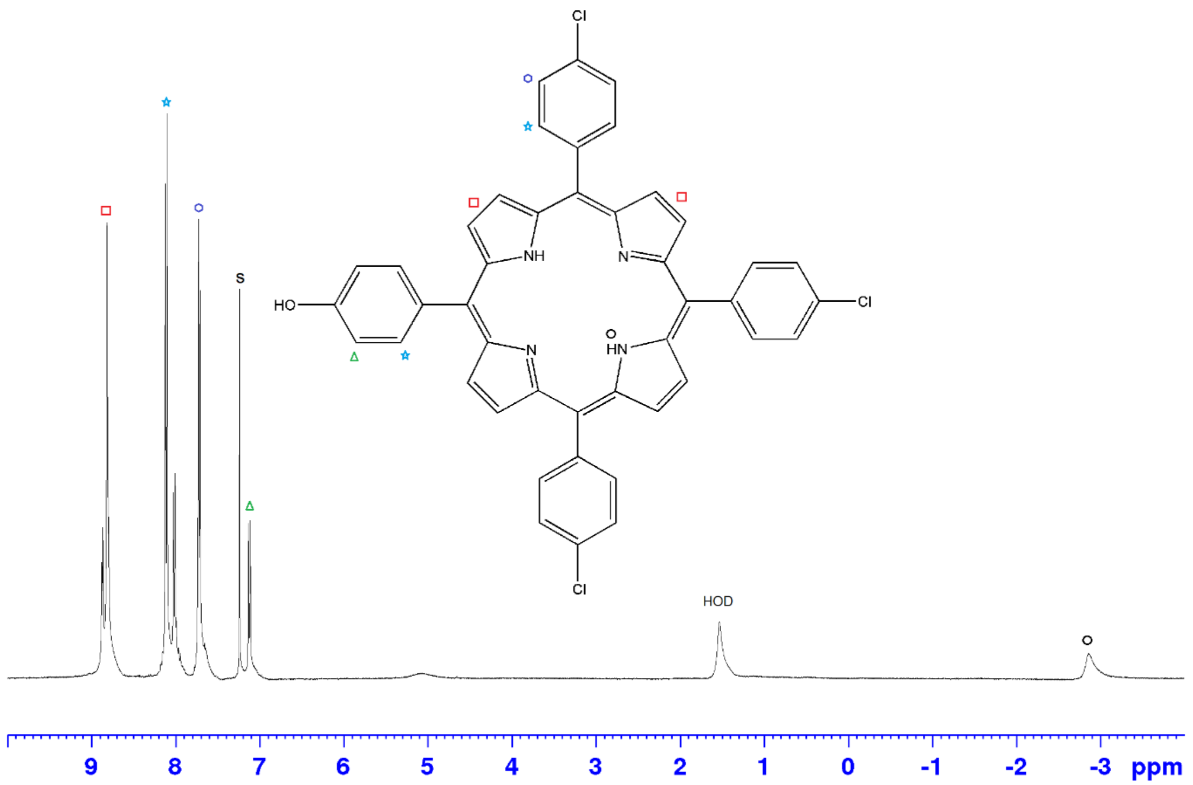

Figure S5. ^{1}H NMR spectrum of *meso*-5-(4-hydroxyphenyl)-10,15,20-tris(4-chlorophenyl)porphyrin $\mathbf{H_2L^5}$ in $CDCl_3$.

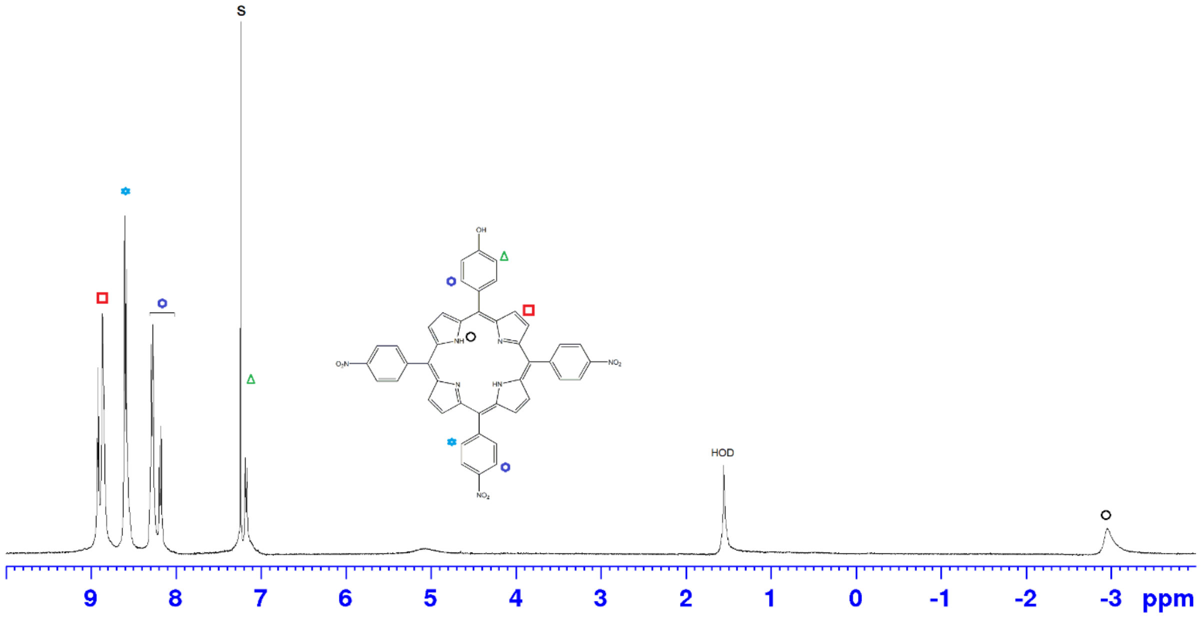

Figure S6. ^{1}H NMR spectrum of *meso*-5-(4-hydroxyphenyl)-10,15,20-tris(4-nitrophenyl)porphyrin $\mathbf{H_2L^6}$ in $CDCl_3$.

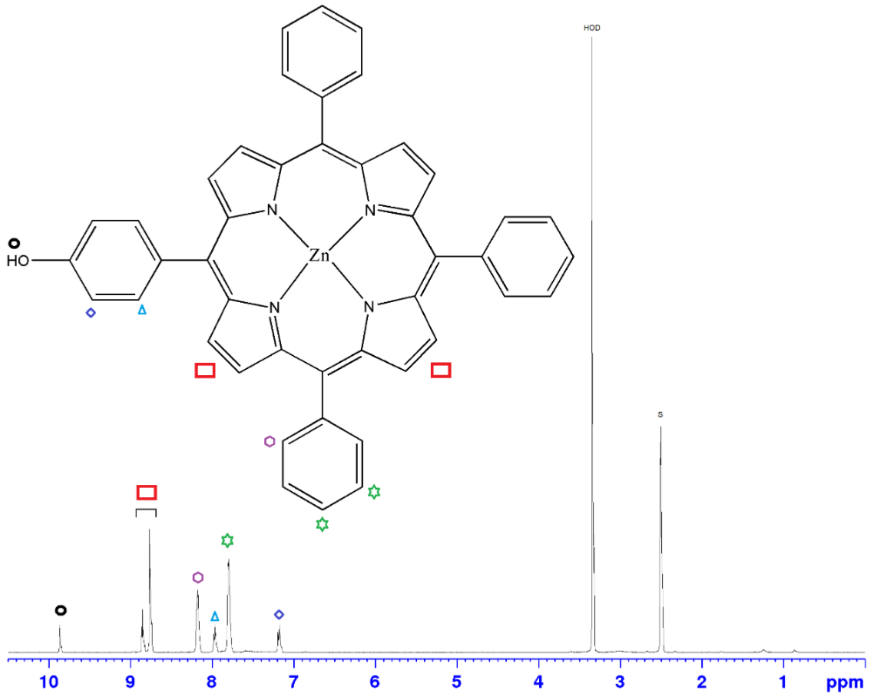

Figure S7. ^{1}H NMR spectrum of *meso*-5-(4-hydroxyphenyl)-10,15,20-tris(phenyl)porphyrinato)zinc(II) **ZnL^{1}** in DMSO-d$_6$.

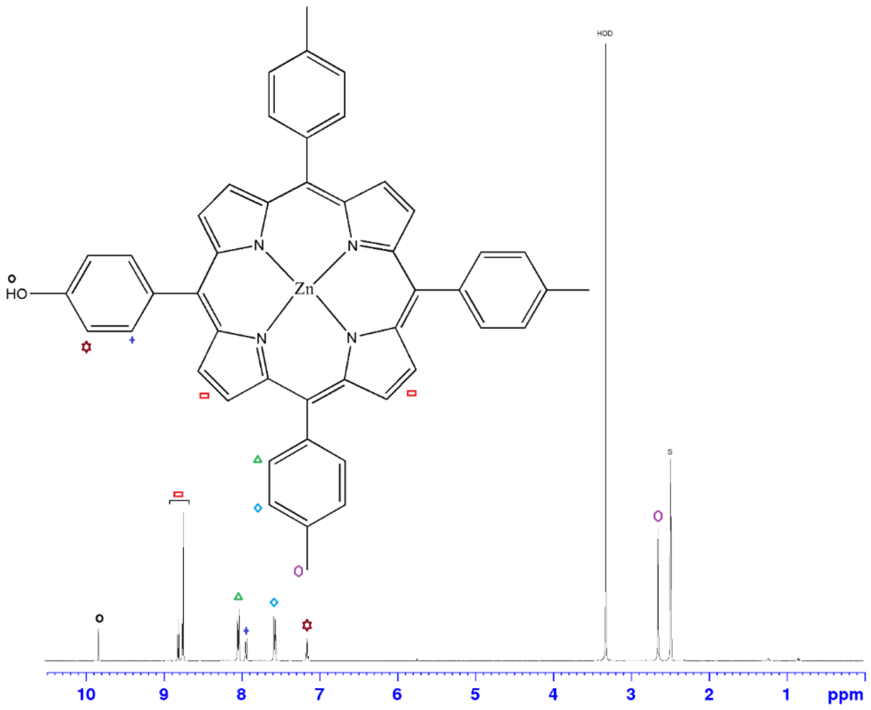

Figure S8. ^{1}H NMR spectrum of *meso*-5-(4-hydroxyphenyl)-10,15,20-tris(4-methyl-phenyl)porphyrinato)zinc(II) **ZnL^{2}** in DMSO-d$_6$.

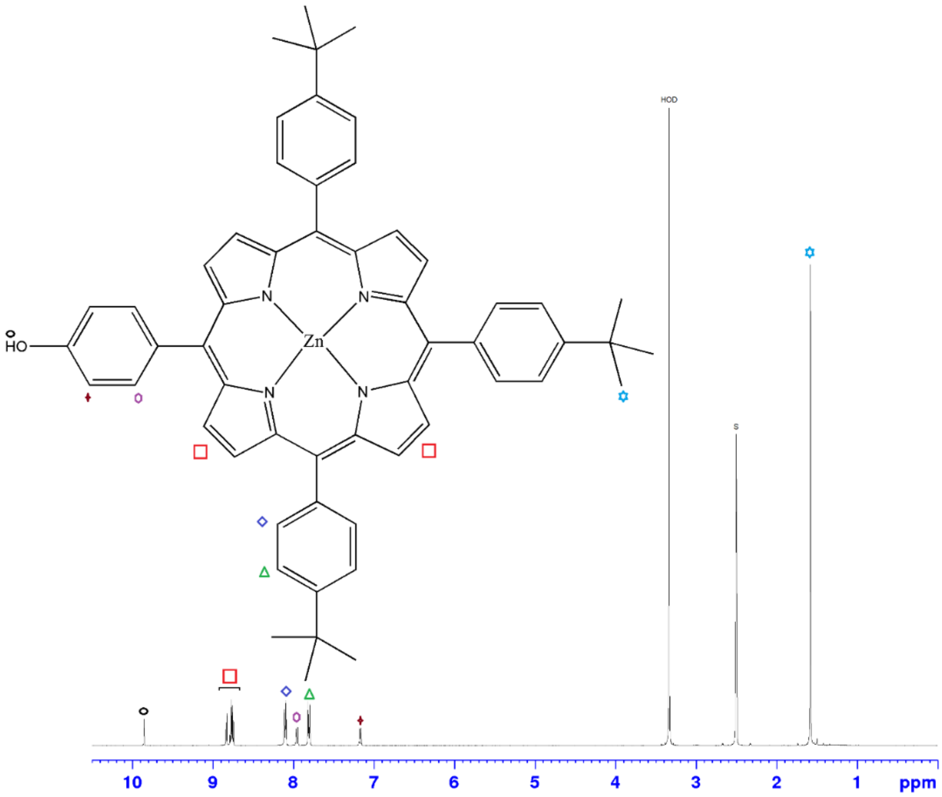

Figure S9. ^{1}H NMR spectrum of *meso*-5-(4-hydroxyphenyl)-10,15,20-tris(4-*tert*-butylphenyl)porphyrinato)zinc(II) **ZnL^3** in DMSO-d$_6$.

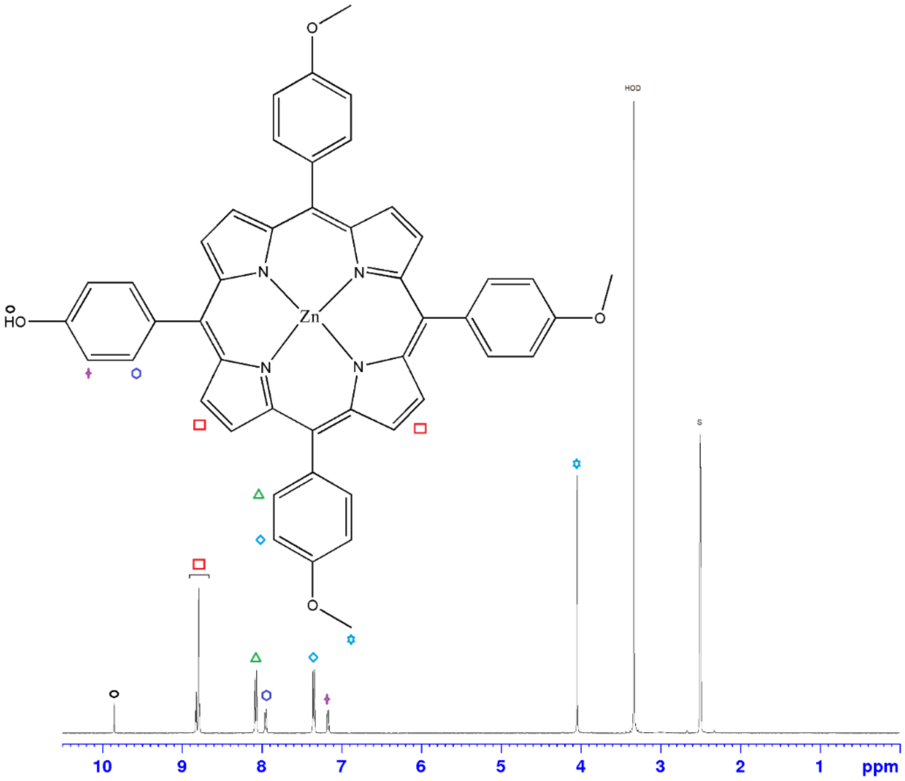

Figure S10. ^{1}H NMR spectrum of *meso*-5-(4-hydroxyphenyl)-10,15,20-tris(4-methoxyphenyl)porphyrinato)zinc(II) **ZnL^4** in DMSO-d$_6$.

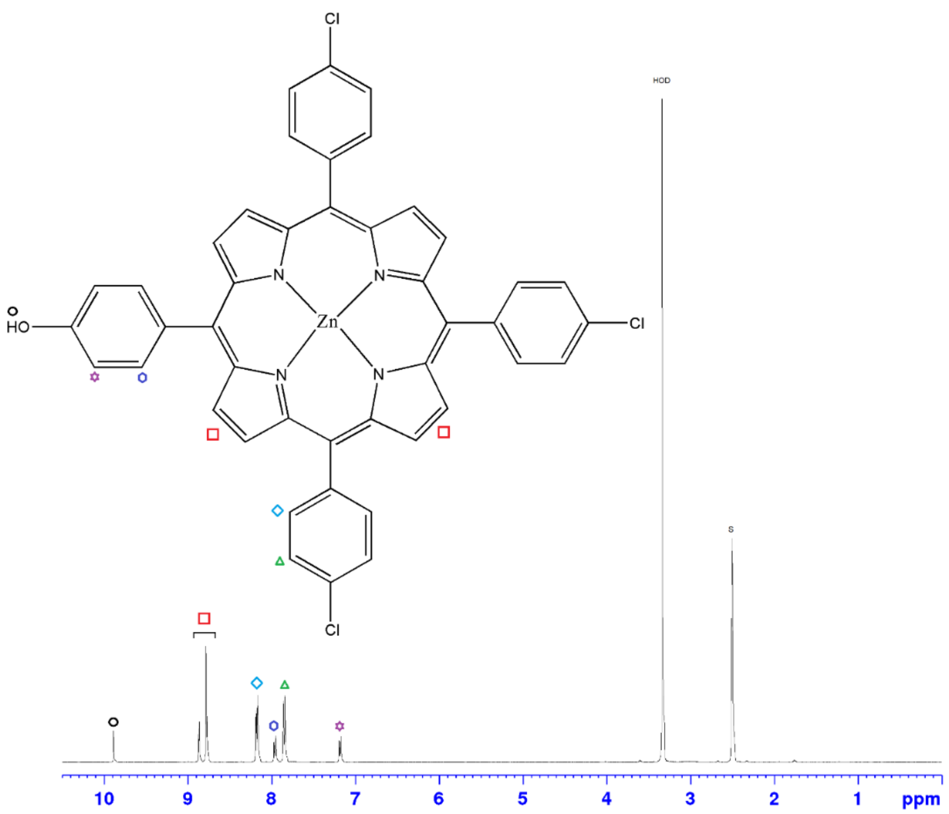

Figure S11. ^{1}H NMR spectrum of *meso*-5-(4-hydroxyphenyl)-10,15,20-tris(4-chloro-phenyl)porphyrinato)zinc(II) **ZnL^{5}** in DMSO-d6.

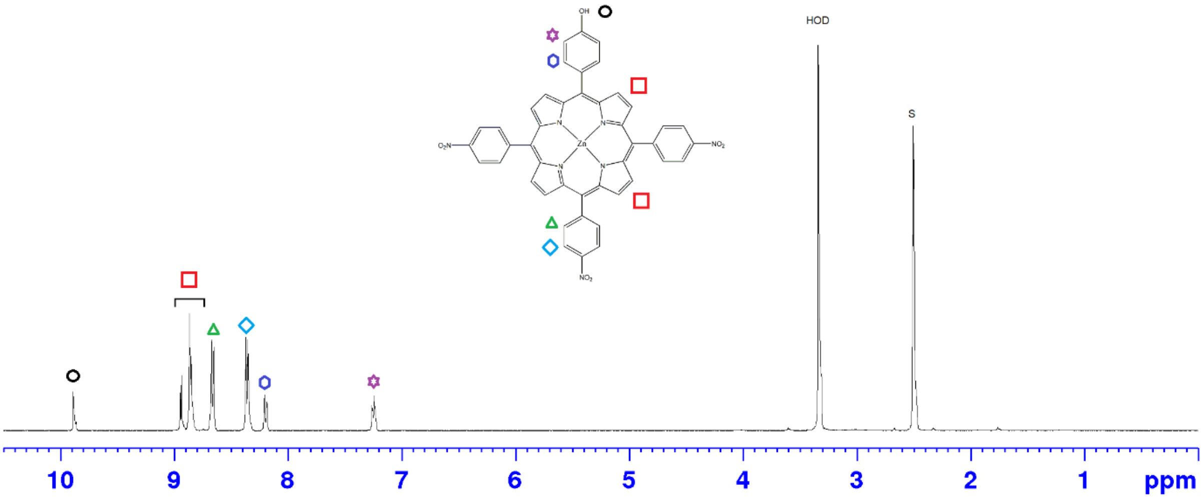

Figure S12. ^{1}H NMR spectrum of *meso*-5-(4-hydroxyphenyl)-10,15,20-tris(4-nitrophenyl)porphyrinato)zinc(II) **ZnL^{6}** in DMSO-d6.

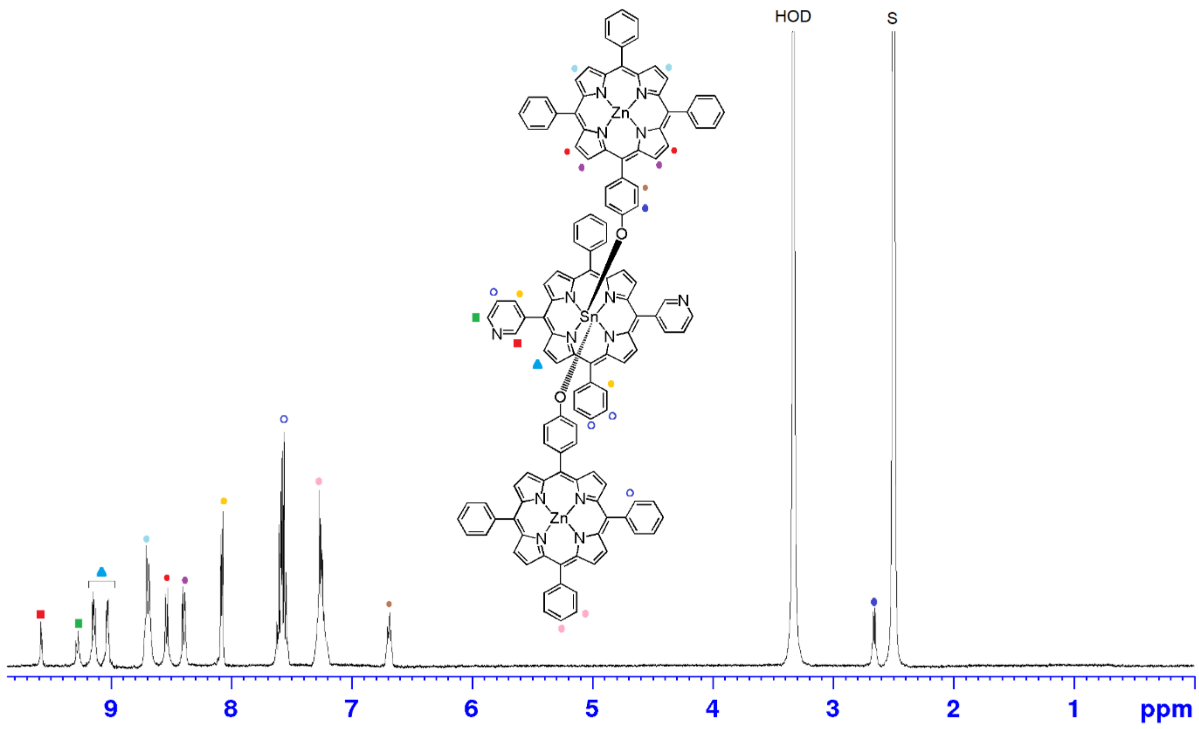

Figure S13. ^{1}H NMR spectrum of triad **1** in DMSO-d$_6$.

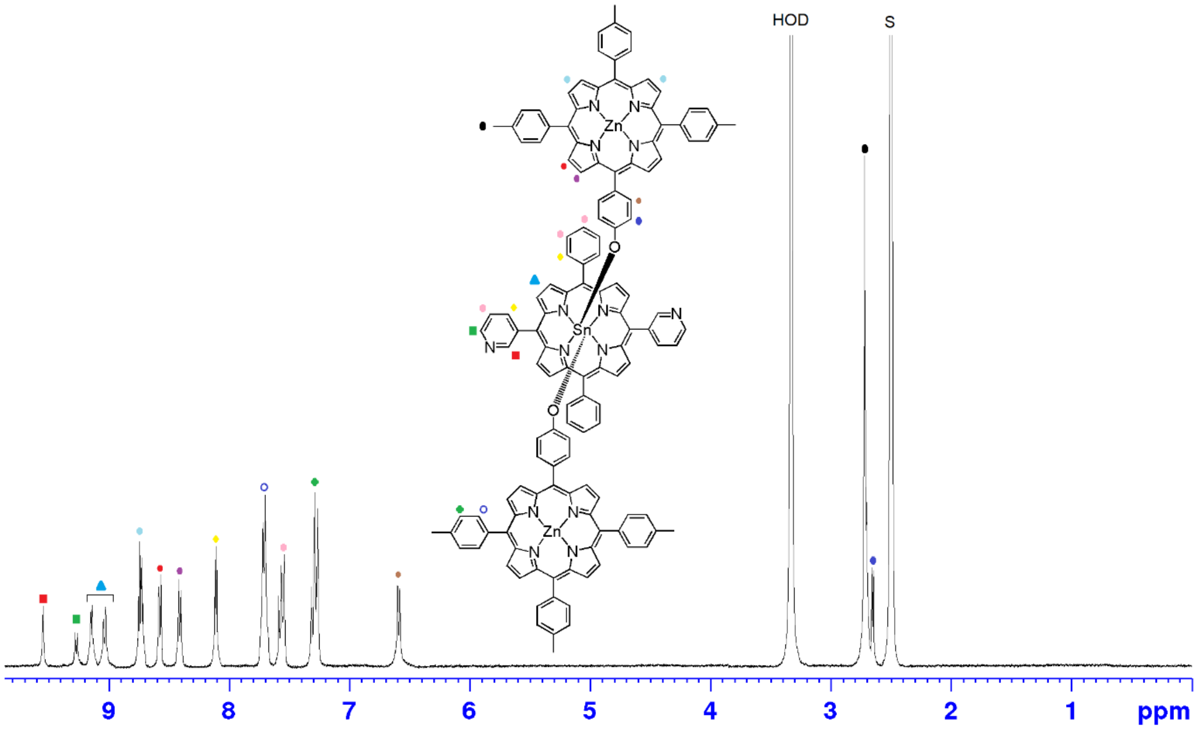

Figure S14. ^{1}H NMR spectrum of triad **2** in DMSO-d$_6$.

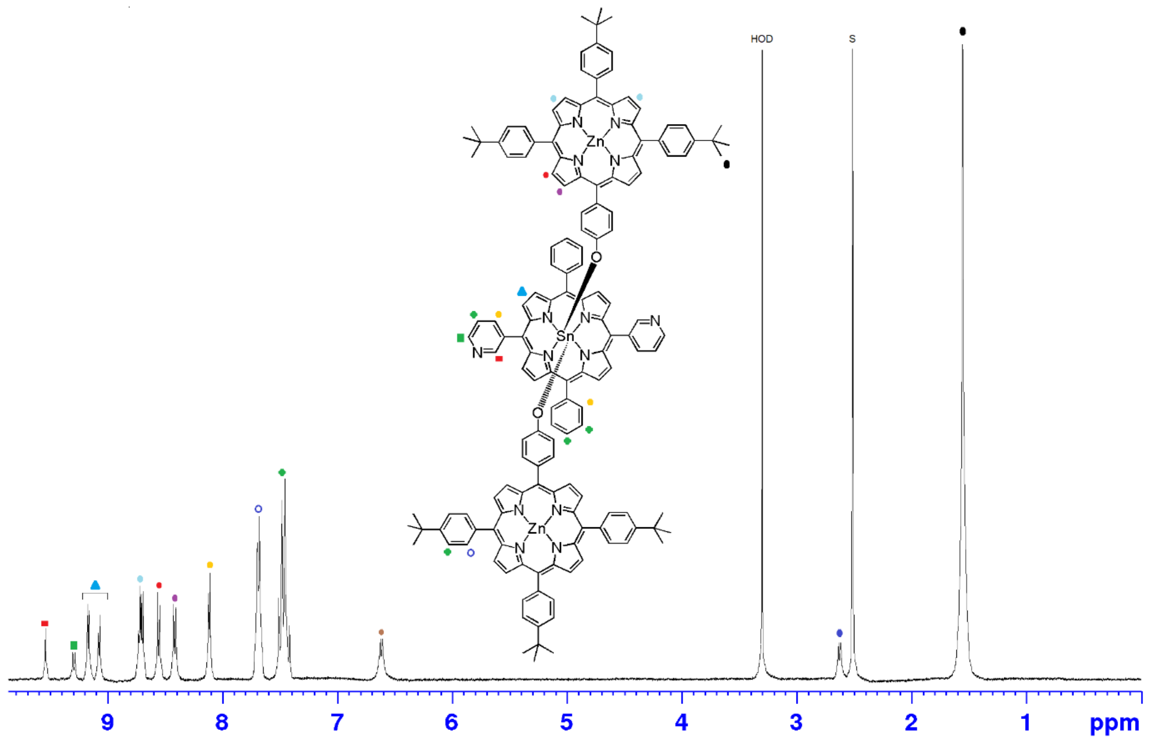

Figure S15. ^{1}H NMR spectrum of triad **3** in DMSO-d6.

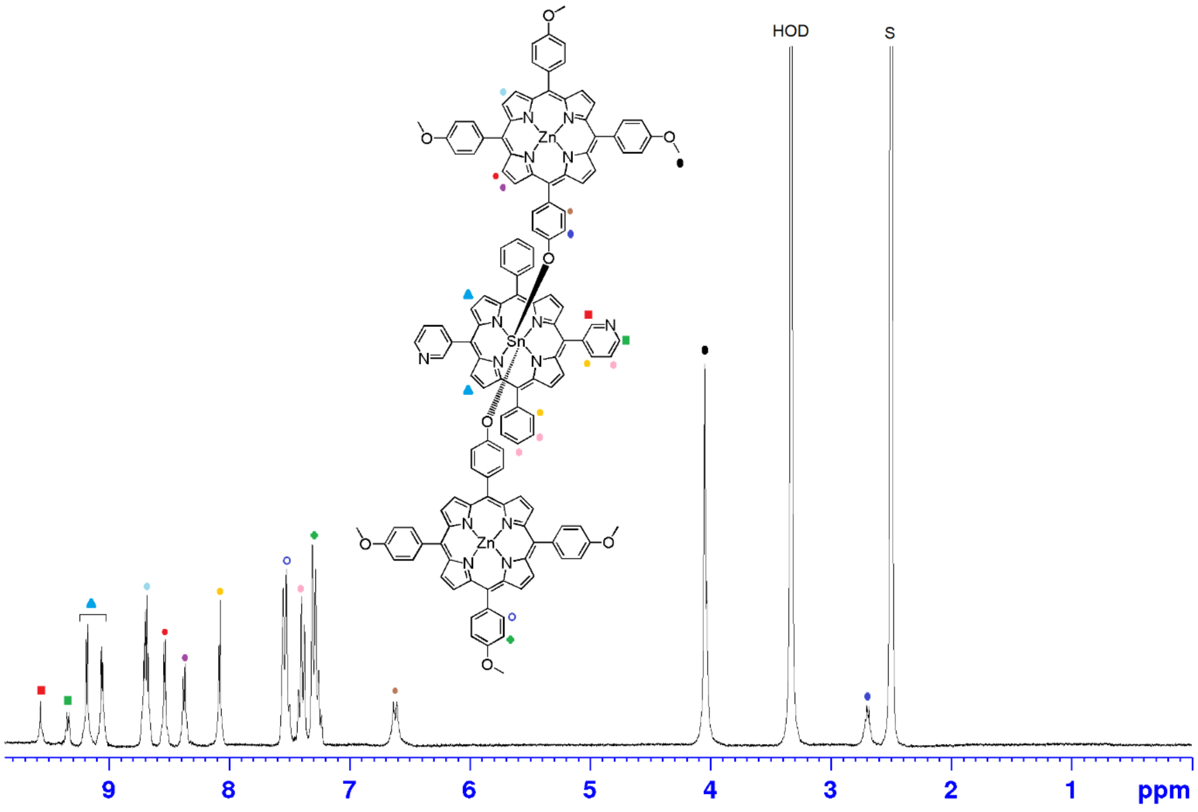

Figure S16. ^{1}H NMR spectrum of triad **4** in DMSO-d6.

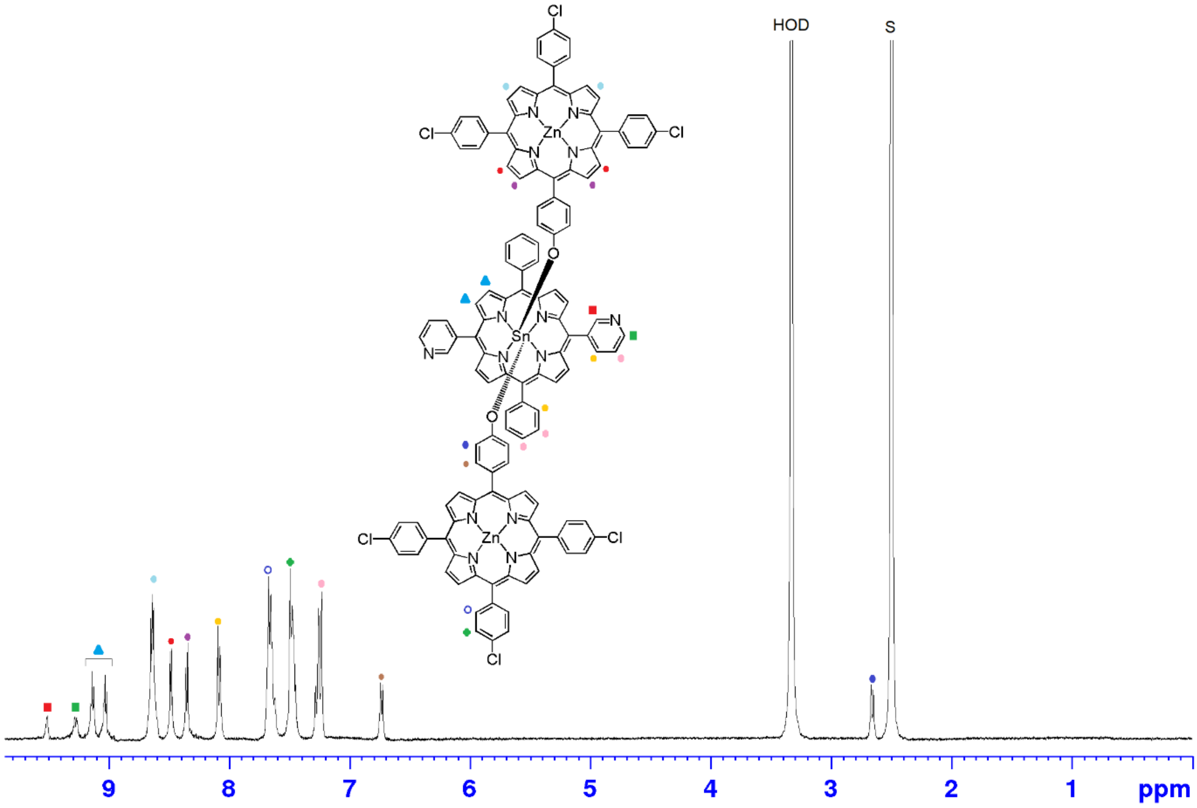

Figure S17. ^{1}H NMR spectrum of triad **5** in DMSO-d6.

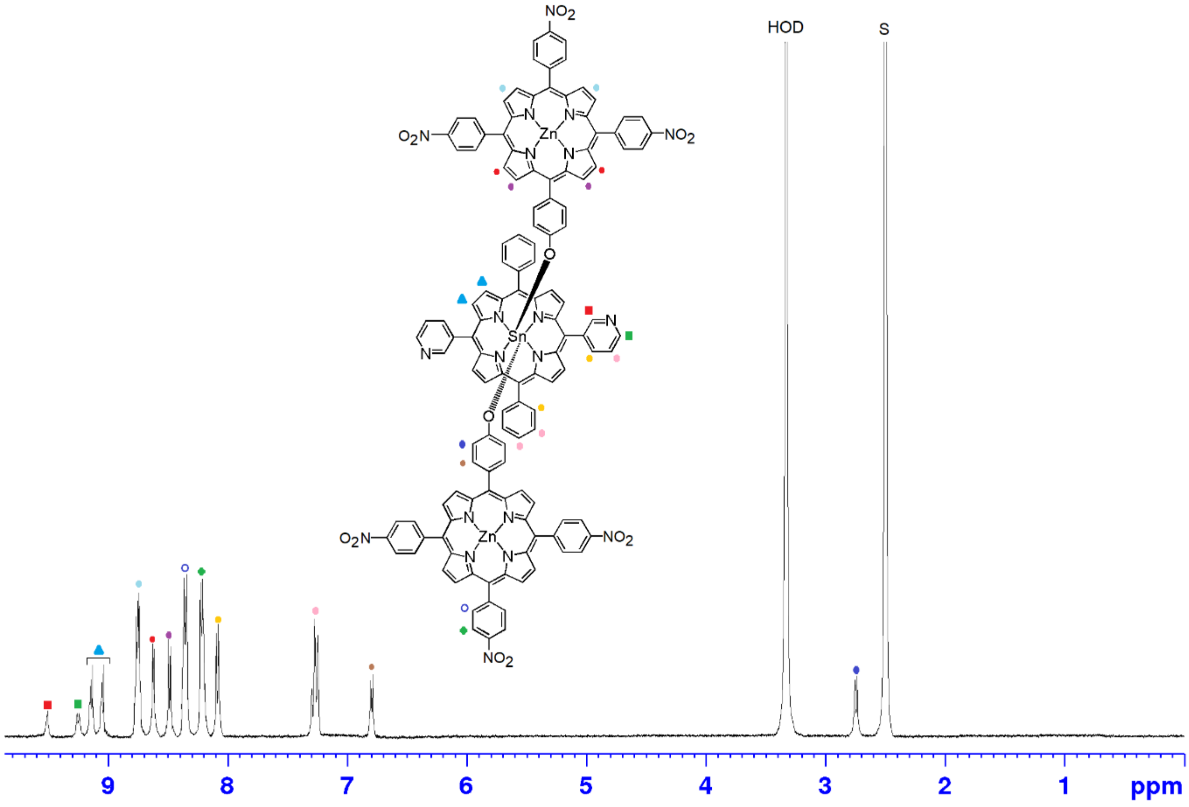

Figure S18. ^{1}H NMR spectrum of triad **6** in DMSO-d6.

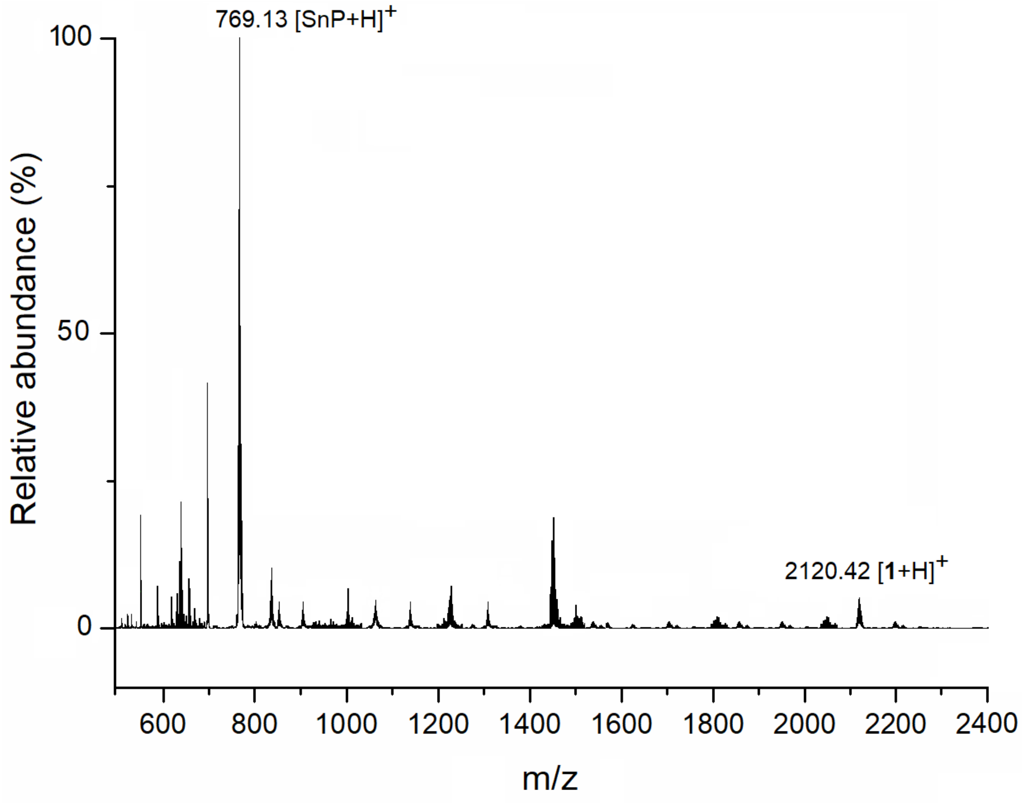

Figure S19. ESI-MS spectrum of triad **1**.

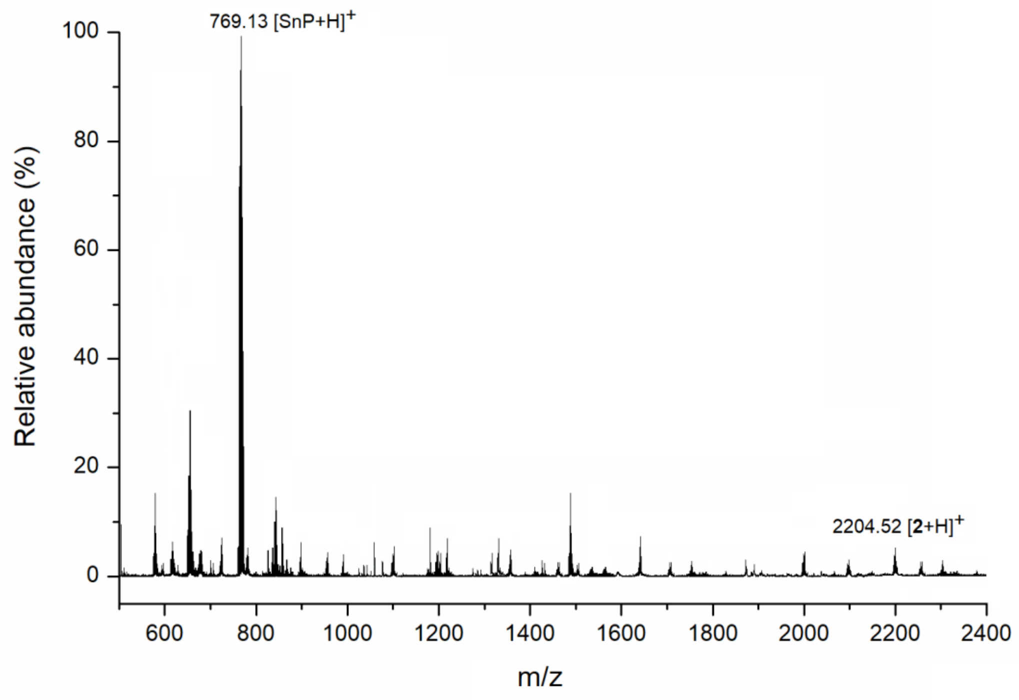

Figure S20. ESI-MS spectrum of triad **2**.

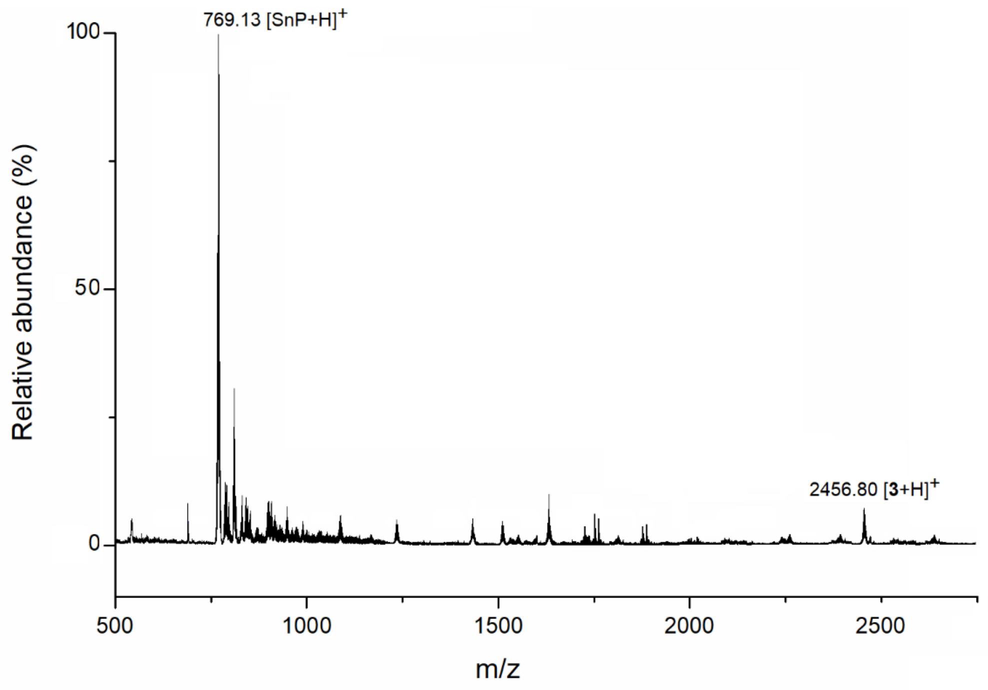

Figure S21. ESI-MS spectrum of triad **3**.

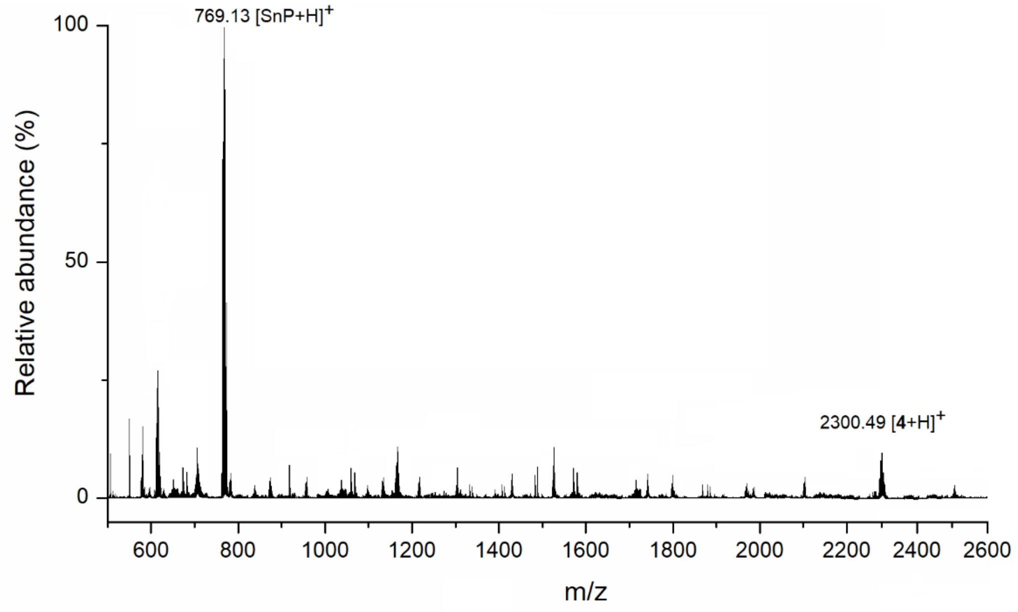

Figure S22. ESI-MS spectrum of triad **4**.

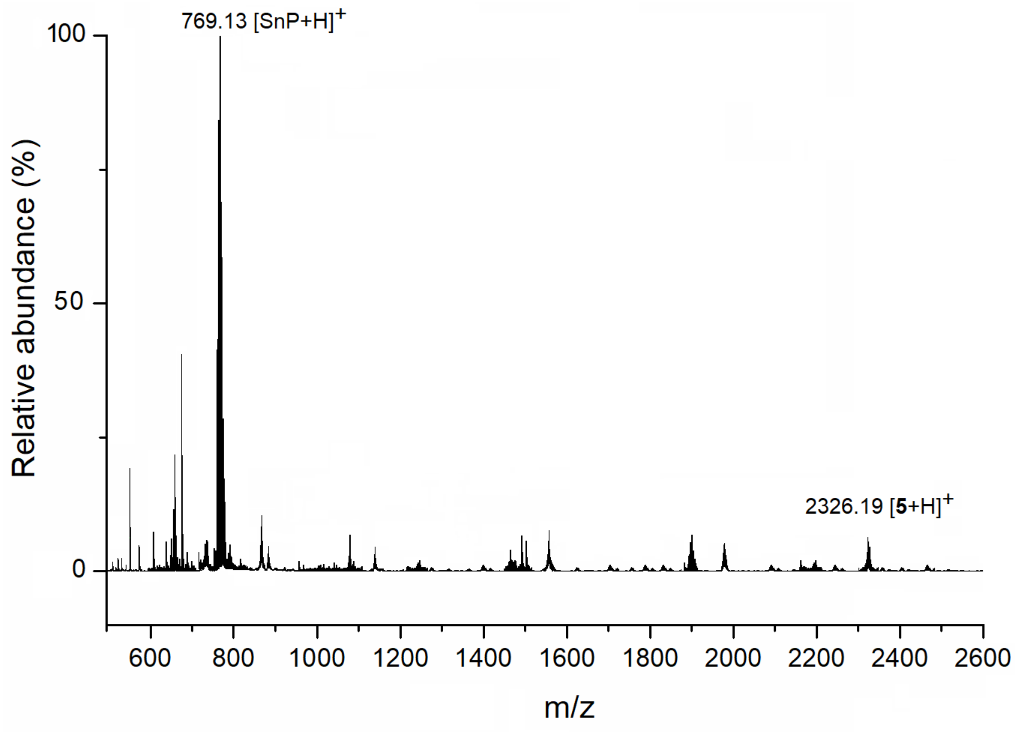

Figure S23. ESI-MS spectrum of triad **5**.

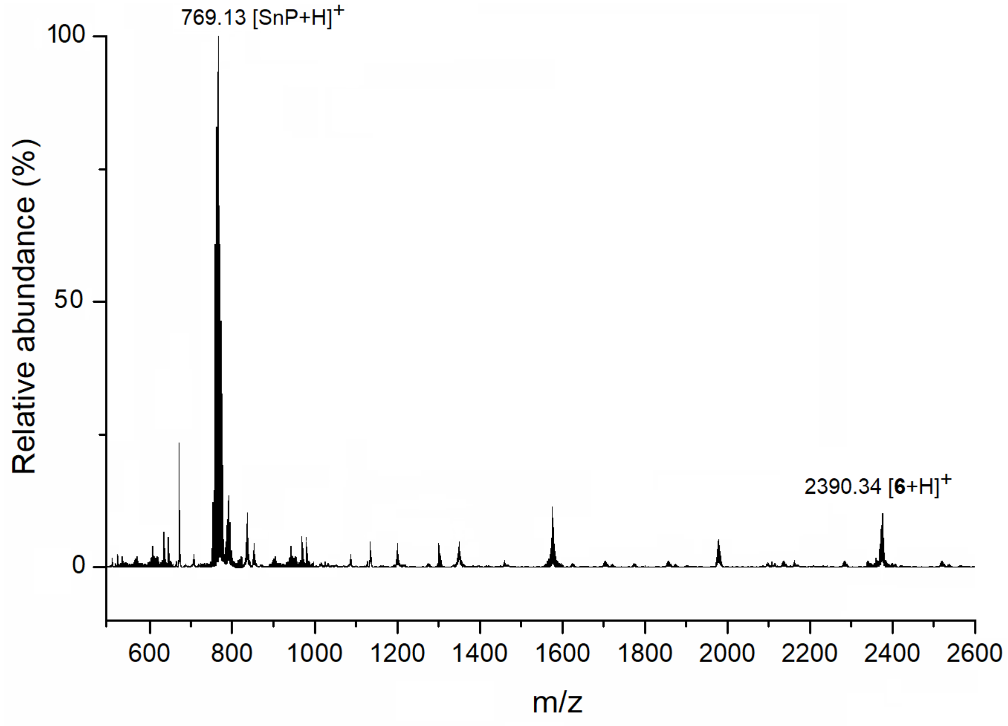

Figure S24. ESI-MS spectrum of triad **6**.

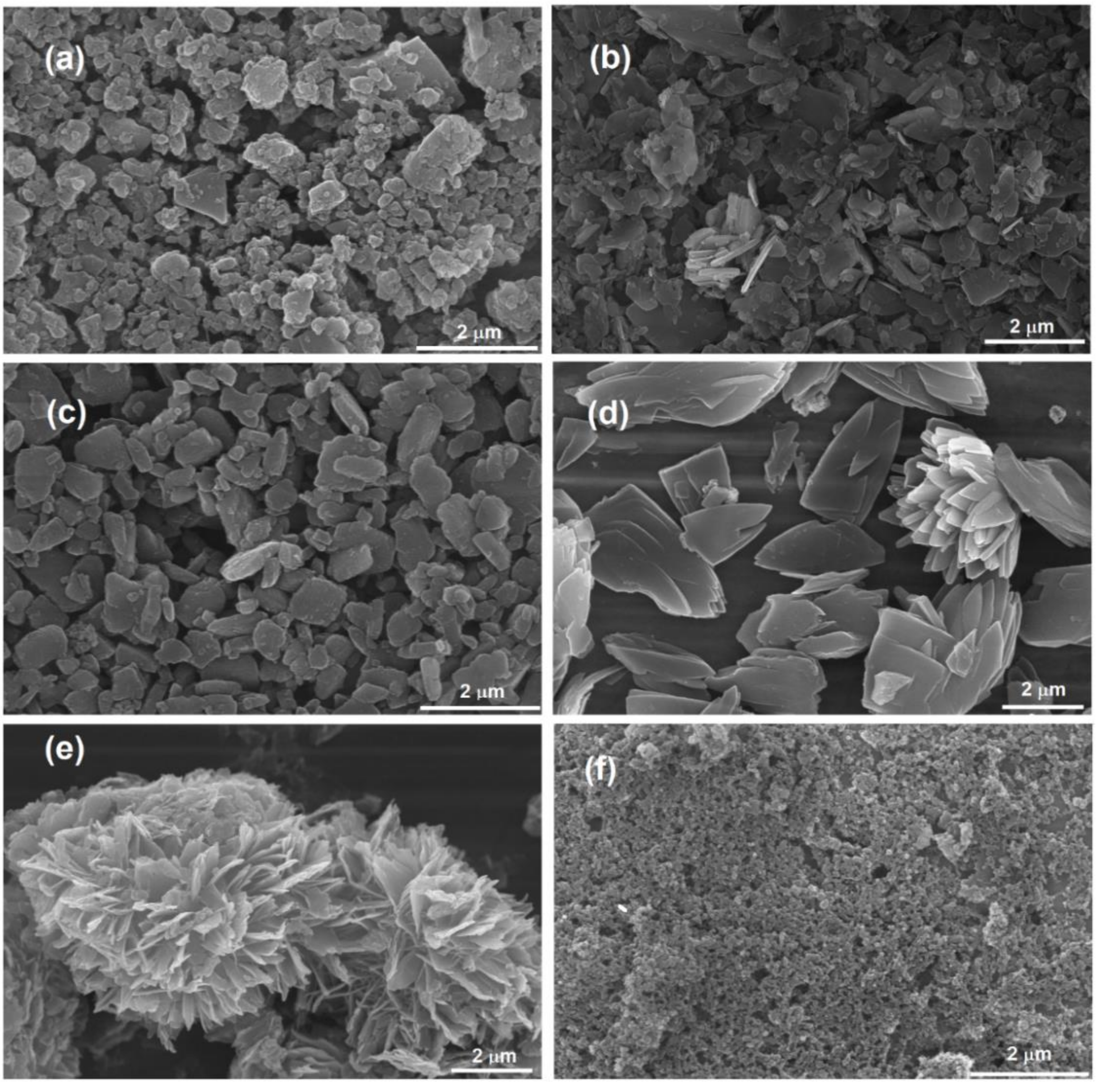

Figure S25. Lower magnification of FE-SEM images for the assembly patterns of triads: (a) **1**; (b) **2**; (c) **3**; (d) **4**; (e) **5**; (f) **6**.

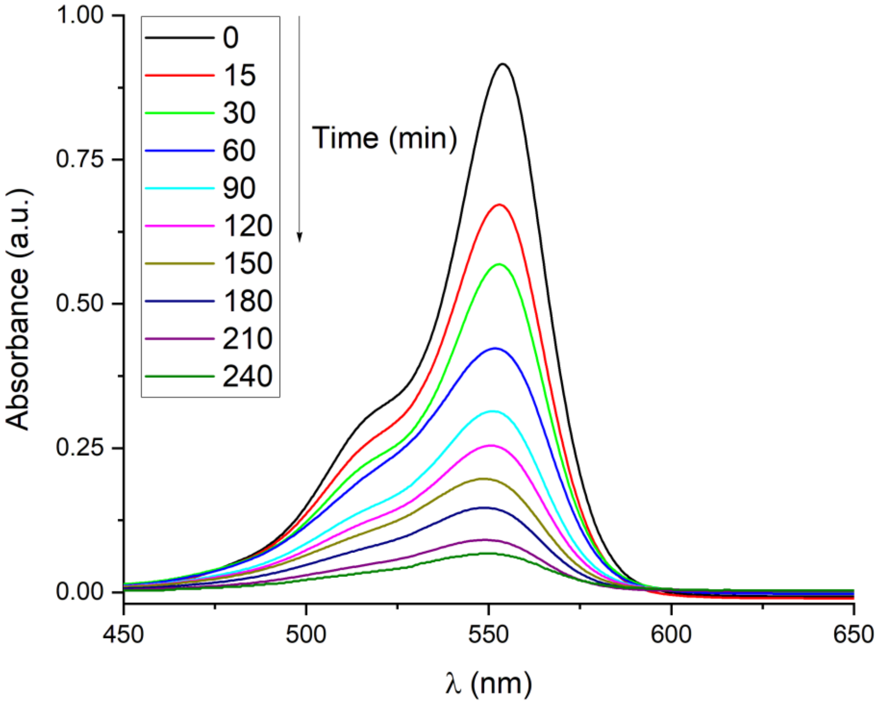

Figure S26. Absorption spectra of RhB in the presence of nano fibers derived from triad **3** under visible light irradiation.

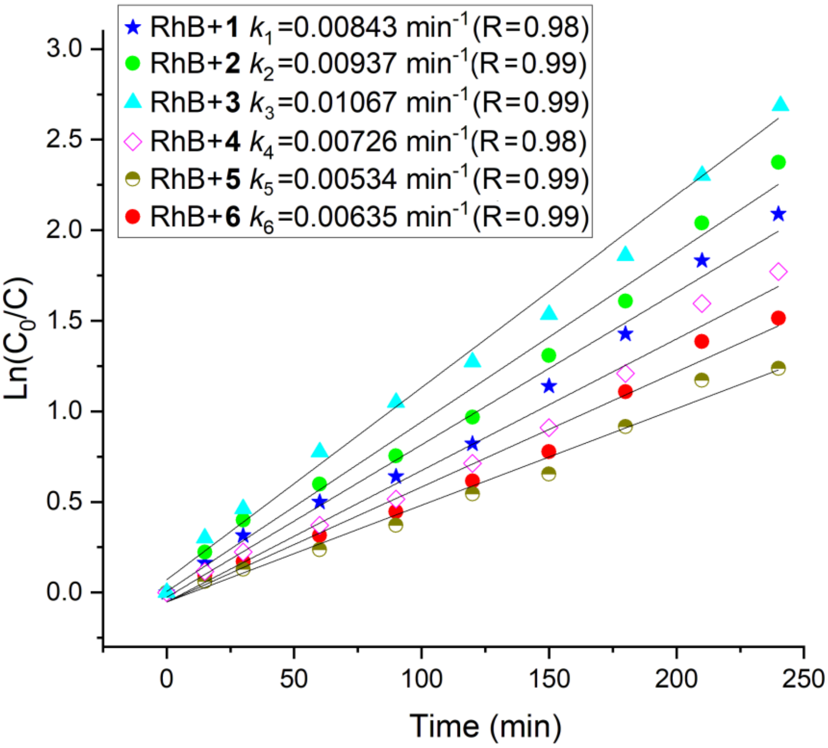

Figure S27. Kinetics for the photocatalytic degradation of RhB under visible light irradiation of the six triads (**1-6**).

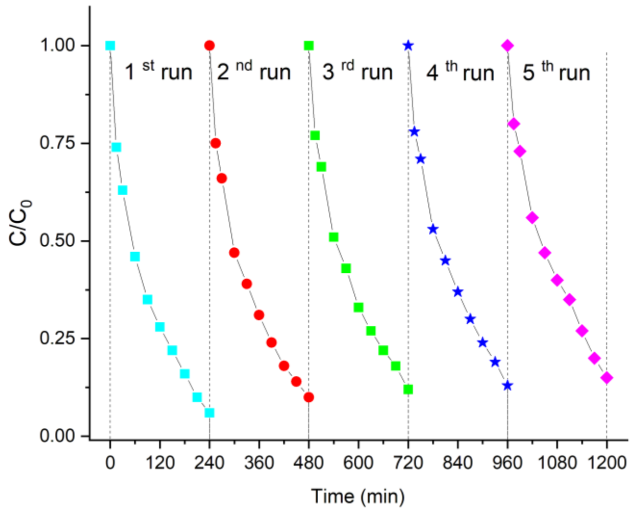

Figure S28. Catalytic cycles (up to 5 cycles) using triad **3** as a photocatalyst for the degradation of RhB.

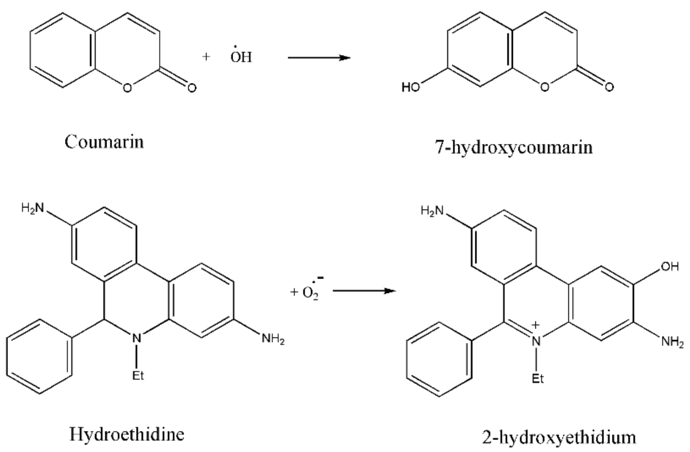

Figure S29. Schematic representation of the detection of the hydroxyl and superoxide radical anion that were generated during the photodegradation experiments.

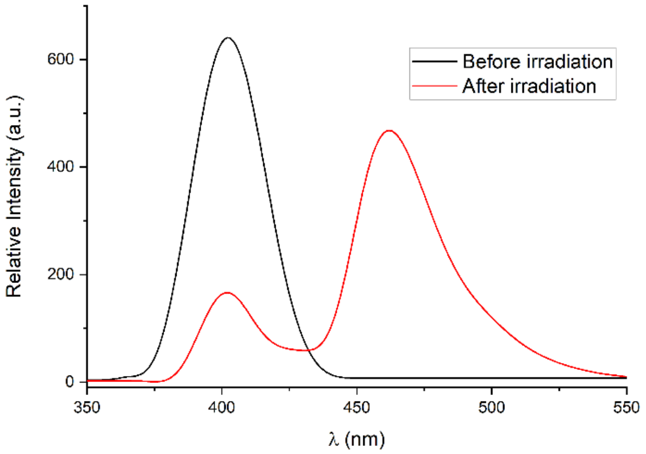

Figure S30. UV-Visible spectra of coumarin in the presence of triad **3** in water. λ_{ex} = 325 nm and light exposure time (30 min).
